# Quantitative Analysis of Repertoire-Scale Immunoglobulin Properties in Vaccine-Induced B-Cell Responses

**DOI:** 10.3389/fimmu.2017.00910

**Published:** 2017-08-14

**Authors:** Ilja V. Khavrutskii, Sidhartha Chaudhury, Sabrina M. Stronsky, Donald W. Lee, Jacqueline G. Benko, Anders Wallqvist, Sina Bavari, Christopher L. Cooper

**Affiliations:** ^1^Department of Defense Biotechnology High Performance Computing Software Applications Institute (BHSAI), Telemedicine and Advanced Technology Research Center, United States Army Medical Research and Materiel Command, Fort Detrick, MD, United States; ^2^Molecular and Translational Sciences, United States Army Medical Research Institute of Infectious Diseases (USAMRIID), Fort Detrick, MD, United States

**Keywords:** repertoire properties, immunosequencing, clonotype, Ebola, statistical analysis, B cell, germinal center, immunoglobulin

## Abstract

Recent advances in the next-generation sequencing of B-cell receptors (BCRs) enable the characterization of humoral responses at a repertoire-wide scale and provide the capability for identifying unique features of immune repertoires in response to disease, vaccination, or infection. Immunosequencing now readily generates 10^3^–10^5^ sequences per sample; however, statistical analysis of these repertoires is challenging because of the high genetic diversity of BCRs and the elaborate clonal relationships among them. To date, most immunosequencing analyses have focused on reporting qualitative trends in immunoglobulin (Ig) properties, such as usage or somatic hypermutation (SHM) percentage of the Ig heavy chain variable (IGHV) gene segment family, and on reducing complex Ig property distributions to simple summary statistics. However, because Ig properties are typically not normally distributed, any approach that fails to assess the distribution as a whole may be inadequate in (1) properly assessing the statistical significance of repertoire differences, (2) identifying *how* two repertoires differ, and (3) determining appropriate confidence intervals for assessing the size of the differences and their potential biological relevance. To address these issues, we have developed a technique that uses Wilcox’ robust statistics toolbox to identify statistically significant vaccine-specific differences between Ig repertoire properties. The advantage of this technique is that it can determine not only *whether* but also *where* the distributions differ, even when the Ig repertoire properties are non-normally distributed. We used this technique to characterize murine germinal center (GC) B-cell repertoires in response to a complex Ebola virus-like particle (eVLP) vaccine candidate with known protective efficacy. The eVLP-mediated GC B-cell responses were highly diverse, consisting of thousands of clonotypes. Despite this staggering diversity, we identified statistically significant differences between non-immunized, vaccine only, and vaccine-plus-adjuvant groups in terms of Ig properties, including IGHV-family usage, SHM percentage, and characteristics of the BCR complementarity-determining region. Most notably, our analyses identified a robust eVLP-specific feature—enhanced IGHV8-family usage in B-cell repertoires. These findings demonstrate the utility of our technique in identifying statistically significant BCR repertoire differences following vaccination. More generally, our approach is potentially applicable to a wide range of studies in infection, vaccination, auto-immunity, and cancer.

## Introduction

Establishing a diverse repertoire of antibody responses is central to humoral immunity acquired through natural infection and vaccination (1–3). Initial diversity arises stochastically from B-cell development, in which naïve B cells are formed following the recombination of discrete variable (V), diverse (D), and joining (J) immunoglobulin (Ig) gene segments into a diverse library of B cells, each expressing its own unique Ig receptor [B-cell receptor (BCR)]. BCR diversity is further increased through affinity maturation within highly specialized compartments called germinal centers (GCs) inside lymphoid tissue, where B cells bind to antigens *via* the BCR and undergo successive rounds of somatic hypermutation (SHM), antigen selection, and proliferation. These GC B cells eventually differentiate into memory B cells and plasmablasts, the latter of which produce a solubilized form of the BCR Ig that is secreted as antibodies (4). Given the importance of antibodies in vaccine-induced immunity, understanding affinity maturation in the context of vaccination will be critical to future vaccine research efforts.

Most of our understanding of GC B cells and affinity maturation is based on the results of studies that have used simple model antigens, such as haptens, which contain a single epitope for BCR binding (5). In such cases, affinity maturation leads to reduced diversity and increased clonality of the GC B-cell repertoire, eventually resulting in a small number of distinct clonotypes, i.e., groups of clonally related B cells, and the emergence of B-cell clones shared across different individuals. Although such studies have yielded deep insights into affinity maturation, these simple model antigens differ greatly from more complex vaccine antigens, such as recombinant proteins, virus-like particles, and whole-pathogen vaccines, which may contain a multitude of epitopes for BCR binding. Hence, it remains unclear whether the oligoclonal responses to model antigens are pertinent to real-world vaccines.

A recent study in mice characterized GC B-cell repertoires generated in response to two complex antigens: influenza hemagglutinin and *Bacillus anthracis* protective antigen (5). The study found that the GC B-cell response to these complex antigens was strikingly different from the responses previously characterized with model antigens. Specifically, affinity maturation resulted in a highly diverse polyclonal response consisting of hundreds to thousands of distinct B-cell clonotypes. This staggering diversity creates a major challenge for characterizing and analyzing vaccine-induced B-cell responses. Because highly polyclonal responses against complex antigens consist of a large number of relatively low-frequency clonotypes, adequate characterization of such responses requires a repertoire-wide approach, in which most, if not all, of the B cells in the repertoire are captured to account for all clonotypes in the response.

We hypothesize that for complex antigens, these highly polyclonal responses result in a systematic shift in repertoire-scale properties away from the naïve repertoire, in an antigen-specific manner. These repertoire-scale properties include features such as V-family usage, the percentage of SHM, or biophysical properties of the third complementarity-determining region (CDR3) of the heavy Ig (IGH) chain principally responsible for antigen binding. Through the use of appropriate statistical analyses, individual or pooled repertoires from different immunization conditions can be quantitatively compared to determine systematic antigen- or vaccine-specific shifts in repertoire-scale Ig properties within the responding B-cell populations.

Next-generation sequencing (NGS) of immune cells, or immunosequencing, provides a means for characterizing complex polyclonal responses on a repertoire scale (1, 2, 6–15). Typically, sequencing is restricted to the IGH or even just its CDR3 region, which is formed by the junction of the V, D, and J gene segments (7, 11, 15, 16).

This approach does not account for the corresponding light chain that makes up the BCR, and is often limited to short sequence reads of ~150 bp. Nevertheless, it provides the necessary sequencing depth to capture the B-cell repertoire in its entirety while containing sufficient sequence information to annotate IGHV and IGHJ genes, establish repertoire lineages, and identify SHMs.

Although NGS is capable of capturing thousands to millions of BCR sequences, extracting statistically meaningful information from such datasets is challenging (8–10, 13, 14, 16). Given the stochastic nature of individual germline repertoires and antigen-specific selective pressures, B-cell repertoires can vary widely depending on the subject, antigen or vaccine conditions, and time (e.g., time postexposure) (17, 18). Despite the fact that various statistical approaches have been applied to analyze B-cell repertoire data, a consensus on the best method remains elusive (2, 8, 12, 19–27).

In many cases, standard statistical approaches, such as the *F*-test or *t*-test, are used to compare differences in means with respect to certain Ig properties in the repertoire. The drawbacks of these approaches are that they (1) often erroneously assume the Ig properties to be normally distributed and (2) can only be applied to properties on an interval scale (e.g., CDR3 length) but not to those on a nominal scale (e.g., VDJ-family usage). Alternate rank-based statistical approaches, such as those of Wilcoxon, Mann–Whitney, and Kruskal–Wallis, can be applied to nominal scale properties (28, 29). However, while such approaches can determine whether two repertoires differ from each other, they are not well-suited to determining the regions where the Ig property distributions significantly differ (18, 30–36) or the confidence intervals for any observed differences.

If the difference in the overall Ig property distributions of two repertoires is deemed to be statistically significant, determining where they differ requires comparing the corresponding proportions for the bins within the distributions. A readily available technique for this purpose is the *z*-test for binomial proportions, in combination with multiple hypothesis testing (see Supplementary Material for details) (28, 29). This is similar to the *t*-test-like techniques and superior to the *F*-test-like techniques, which was used in previous studies without any corrections for multiple hypothesis testing (37–39). However, both the *z*-test and the *F*-test-like techniques come with their own set of assumptions—for example, that the distribution of the proportions is nearly normal (28, 29)—and cannot provide the confidence intervals that are necessary to determine the magnitude of the observed effects and to help judge whether the effects are potentially biologically relevant.

Statistical methods, such as the Storer–Kim (SK) (40) and Kulinskaya–Morgenthaler–Staudte (KMS) (41) tests have recently been developed to identify statistically significant differences between distributions (42) without making any assumptions about their normality. Although the SK test is one of the most powerful non-parametric tests, it does not provide confidence intervals (43). Therefore, here we combine the SK test with the KMS test, which also provides confidence intervals. Together, these tests can be used not only to identify statistically significant differences between BCR repertoires but also to provide confidence intervals that are useful for assessing the magnitude of the observed effects and their potential biological relevance. We emphasize that the main advantage of these methods is that they make no assumptions regarding the shape of the underlying distributions and can be applied to non-normal or irregular distributions. However, as do many statistical tests, the SK and KMS methods assume that each observation in the dataset is independent.

The assumption of independence of observed sequences in immune repertoires is likely to be erroneous because affinity maturation creates clonally related BCR sequences that share a common ancestor and have inherent parent–child relationships. Clonally related sequences with the same parent (i.e., the rearranged VDJ germline sequence) but different SHMs are part of the same clonotype. Previous studies have used clonotype clustering to identify clonally related sequences on the basis of sequence similarity and collapse the sequence dataset to a list of clonotypes that can be assumed to pass the criteria of independence (2, 23–25). However, evaluation of repertoire properties at the clonotype level presents some additional challenges.

Some Ig properties, such as CDR3 length or V-family usage, are well-defined at the clonotype level because all members of the clonotype family share that same property. However, other Ig properties, such as SHM percentage or biophysical characteristics, are not easily defined at the clonotype level because they vary across members within the clonotype family. Previously, property distributions at the clonotype level were derived from the properties of representative sequences, one per clonotype (44, 45), which may be subject to arbitrary selection criteria or PCR amplification and sequencing errors (46). Here, we introduce a more general approach based on a weighted-average property of all sequences within the clonotype. In this approach, we use sequence reads or template counts normalized within each clonotype to avoid intrinsic biases and minimize the influence of sequencing errors on the results.

The Ebola (EBOV) virus-like particle (eVLP) vaccine candidate can provide protection against lethal viral infection in mice, guinea pig, and non-human primates (47, 48). eVLPs, which spontaneously form through co-expression of EBOV VP-40 matrix protein and glycoprotein (GP), represent a highly complex vaccine with antigen display and structural properties identical to those of infectious EBOV particles. Recently, we have demonstrated that acute protective immune responses mediated by eVLP vaccination are associated with robust T-cell and antibody responses; however, inclusion of adjuvants is necessary to confer durable immunity (>5 months) in mice (49, 50). Specifically, the clinical grade RNA adjuvant, polyinosinic-polycytidylic acid (poly-IC)-poly-l-lysine carboxymethylcellulose [poly-ICLC (pICLC)], augments eVLP-mediated humoral immune responses, and GC B-cell reactions (50, 51). However, BCR repertoire analysis of these GC B-cell responses has yet to be defined, and Ig repertoire-level characterizations of eVLP-mediated B-cell responses have been limited (21).

In this study, we characterized the early GC B-cell dynamics following single-dose eVLP immunization in mice, under both non-adjuvant and adjuvant conditions. We used NGS to define the GC B-cell repertoires under different vaccine conditions, and compared them with naïve B-cell repertoires from unvaccinated subjects. In particular, we applied a statistical approach, based on the SK and KMS methods, to analyze the Ig repertoire property distributions and to determine *where* the distributions differ statistically significantly. This approach allowed us to identify robust antigen-specific repertoire features based on a range of Ig properties, including V-family usage, SHM percentage, CDR3 length, and other CDR3 biophysical properties. Finally, we explored group- and individual-level variation, combined with down-sampling of the repertoires, to determine the robustness of the defined antigen-specific repertoire features across individuals.

## Materials and Methods

### Overall Approach

Here, we present a new statistical analysis approach for comparing B-cell repertoires that develop in response to different antigen or immunization conditions. The main goal is to identify statistically significant features in the repertoires that capture robust differences in antigen immunization conditions. In this analysis, a repertoire can be derived from a single subject or pooled from multiple subjects with identical or similar immune or antigen conditions. We applied this approach to a well-characterized eVLP-based vaccine candidate with proven efficacy in multiple animal models, including mice (50, 52).

**Figure 1:**
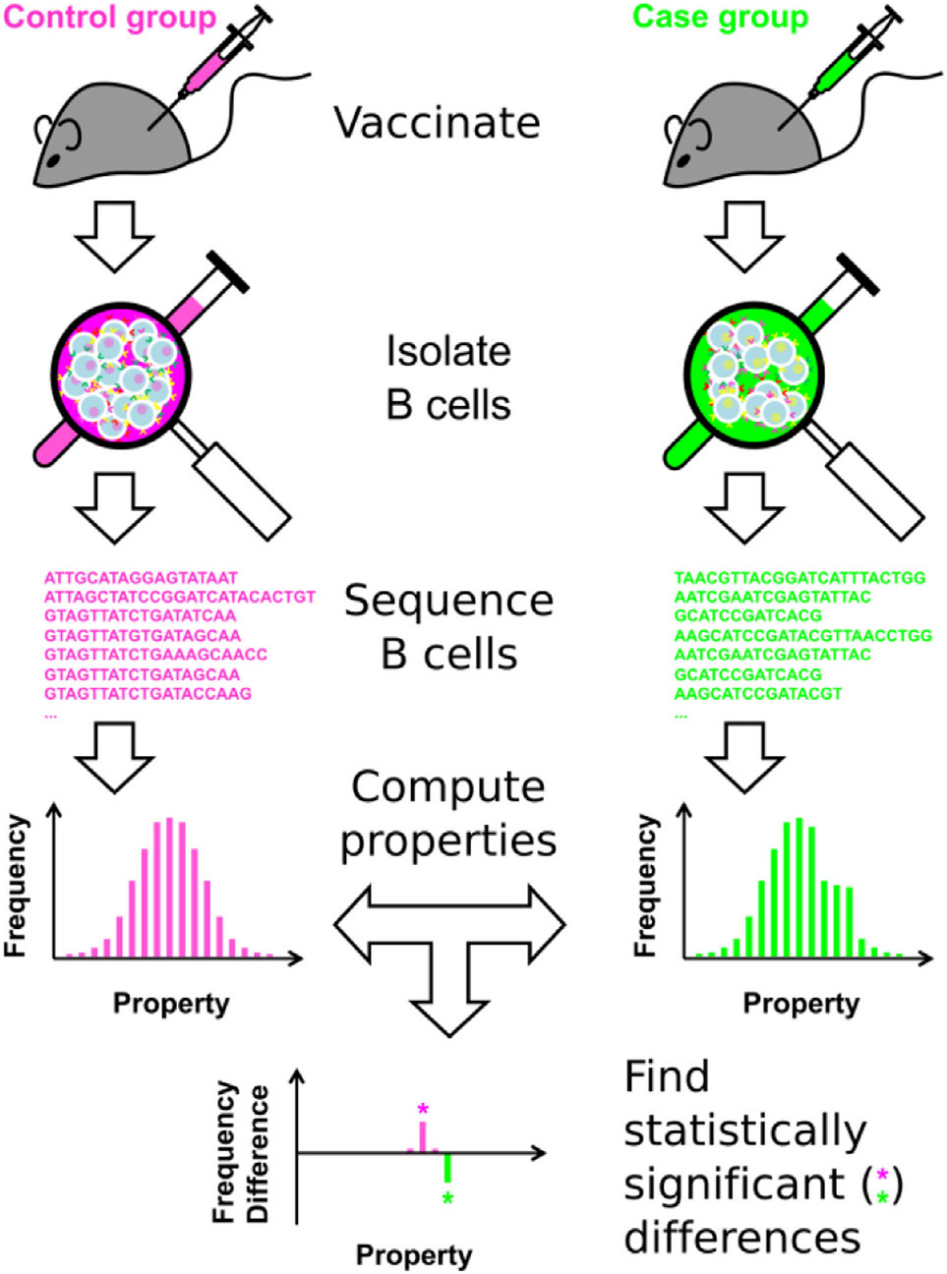
Overview of the approach.

The overall approach is shown in Figure 1, and summarized here. (1) Following immunization, B cells are isolated from a subject or population of interest. These B cells can be isolated from peripheral blood mononuclear cells, or directly from lymphoid organs, such as the lymph node or spleen, in animal models. (2) Immunosequencing, of which there are many types [reviewed in Ref. (8, 12, 19, 25–27)], is carried out on the B-cell sample. Here, we carried out bulk sequencing of the murine IGH BCR locus, focusing on the CDR3 region. The approach can also be applied to any type of heavy chain or paired heavy chain/light chain BCR sequencing. (3) For each unique BCR sequence, a battery of Ig properties is calculated, including nominal scale (e.g., V-family usage), discrete interval scale (e.g., CDR3 length), and continuous interval scale properties (e.g., SHM percentage). (4) Clonotyping is performed to cluster the clones into distinct, independent clonotypes, in order to ensure the assumption of independence necessary for statistical analysis. The properties of all Ig sequences are weight-averaged, using individual sequence reads within the clonotype to minimize the effect of sequencing errors and PCR amplification bias. (5) Statistical analysis is then performed, using a consensus of the SK and KMS methods. This approach represents each repertoire as a set of histograms for each Ig property, and determines where the two repertoires significantly differ at the clonotype level.

### Vaccination

Ebola virus-like particles were produced as previously described (52). Briefly, 293T cells were co-transfected with EBOV (Kikwit) GP_1,2_ and matrix protein, VP40. VLP supernatants were collected at 72 h post-transfection and purified by sucrose gradient centrifugation and their GP contents were determined by western blotting. To ensure sterility, eVLPs were irradiated at 10^6^ rad; they contained less than 25 EU/ml endotoxin and less than 10 colony-forming units of bacteria per vaccination. VLPs were maintained at −80°C and diluted in sterile saline and/or combined with pICLC prior to vaccination. pICLC (Hiltonol^®^) was provided by Oncovir, Inc. (Washington, DC, USA).

C57BL/6 mice (6–8 weeks in age) were obtained from Jackson Labs (Bar Harbor, MA, USA). The subject mice were vaccinated with eVLP (10 µg GP_1,2_ content) alone or in conjunction with pICLC (10 µg) by intramuscular injection. Animal work was performed under a protocol approved by the USAMRIID Institutional Animal Care and Use Committee, in compliance with the U.S. Animal Welfare Act, Public Health Service Policy, and other federal statutes and regulations relating to animals and experiments involving animals. The facility where this research was conducted (USAMRIID) is accredited by the Association for Assessment and Accreditation of Laboratory Animal Care International, and adheres to the principles stated in the Guide for the Care and Use of Laboratory Animals (National Research Council, 2011).

### Experimental Characterization of Immune Responses

#### Enzyme-Linked Immunosorbent Assay (ELISA)

Enzyme-linked immunosorbent assays were performed as previously described (50). In brief, blood was collected from vaccinated mice at the indicated time points in vacutainer serum-separating tubes. ELISA plates were coated with recombinant mammalian EBOV GP_1,2_ at 2 µg/ml in phosphate-buffered saline (PBS; Corning Life Sciences, Corning, NY, USA). Sera were diluted by half-log dilutions starting at 1:100, and incubated for 1 h on GP_1,2_-coated plates. The plates were washed and then incubated with the indicated secondary horseradish peroxidase antibody. ELISAs were developed using 3,3′,5,5′-tetramethylbenzidine substrate/stop solution and measured on a Tecan plate reader. The absorbance cut-off was determined as background +0.2 U of optical density.

#### Flow Cytometry

Single-cell suspensions of draining lymph nodes (dLN) were collected at the indicated time points. Cells were washed with FACS buffer [PBS, 0.5% BSA and 2 mM EDTA; Corning (Corning, NY, USA), Sigma (St. Louis, MO, USA)], lysed with red blood cell buffer (Sigma), and subsequently counter-stained with the following B-cell antibodies: B220, IgM, IgD, CD38, CD95, and GL7 (all purchased from Becton-Dickinson Biosciences, BD, Franklin Lanes, NJ, USA). All samples were Fc-blocked (anti-CD16/CD32, BD), and stained to evaluate viability (live/dead aqua, Invitrogen) prior to antibody staining. Data were collected on a BD FACS Canto II or BD Aria II and analyzed with FlowJo (Tree Star).

### Sample Preparation and Sequencing

Highly purified (>95%) day 10 (D10) postvaccination GC B cells (B220^+^GL7^+^CD95^+^) from the popliteal lymph nodes were isolated by cell sorting on a BD Aria II (*n* = 6). To serve as a baseline control, naïve B cells (B220^+^IgM^+^IgD^+^GL7^−^CD95^−^) were sorted from unvaccinated control mice (*n* = 2). Subsequently, purified B-cell populations were pelleted by centrifugation and snap frozen on dry ice. The frozen samples were then shipped to Adaptive Biotechnologies (Seattle, WA, USA) for DNA extraction and NGS of the murine BCR heavy chain VDJ loci. The final BCR sequences were obtained from amplicons spanning the framework region 3 (FR3) of the Ig heavy chain variable (IGHV)-gene segment to the 3′ end of the complete VDJ junction. Each sequence was trimmed to 125 bp, maintaining the last 3 nt of the sequence codes for the conserved 118 Trp (TGG) of the CDR3 region (16).

### Data Preprocessing

The immunosequencing data provided by Adaptive Biotechnologies (16) were organized in a table, with each entry occupying a row and columns containing the 125-bp sequence, the V, D, and J gene segment annotations, numbers of deletions and insertions, and number of mutations in the V gene segment as identified by the international ImMunoGeneTics (IMGT) information system (53, 54). Each entry included the amino acid sequence and length of CDR3 loop and was identified as productive or non-productive.

We assembled an SQL database to store and analyze the sequence data. In this study, we loaded a total of 61,327 sequences into the database. We extracted all sequence entries labeled as “In” (for in-frame or productive), and which had annotated V and J segments. We next removed any additional sequences that had stop codons. The resulting database contained 42,606 sequences. No additional filters were applied.

### Clonotype Clustering

Clonotype clustering is a common approach for grouping clones in a manner that ensures, to a first approximation, the independence of observations necessary to carry out a statistical analysis (8, 9, 25, 44, 53, 55, 56). We used a single-linkage pairwise clustering approach, which groups B-cell clones into a clonotype family, by finding BCR sequences with matching IGHV-gene annotation and CDR3 lengths but differing only by a single mismatch in the CDR3 amino acid sequence. As in any clonotype clustering approach, sequences from two different clonotypes that are very similar may be pooled together, while those from the same clonotype may be split into several clonotypes if the repertoire is sparse.

We developed an in-house Perl script for clonotype clustering. First the program ordered all sequences by group, subject, CDR3 length, and decreasing template count. Starting from the sequence with the highest template count, it constructed a network of sequences by adding sequences with the same group and subject identifiers with matching IGHV-gene segment annotations, and CDR3 lengths that differed from one of the sequences already in the network by at most one amino acid in the CDR3 loop, until no further sequences could be added to the current network. Once the program added all such sequences, it moved on to the next sequence with the highest template count, and the procedure was repeated until all sequences had been assigned to their networks. Each completed network defined a clonotype. Finally, for each clonotype, we computed the number of members using unique sequences and the aggregate number of template counts for all sequences that comprised the clonotype. Note that clonotype clustering was performed at the subject level, after which the identified clonotypes from each subject were pooled into corresponding groups.

### Clonality and Diversity Scores

To compute the clonality scores of the sample, we used a measure based on normalized entropy (57). Because this measure does not depend on the number of observations in the sample, it can be applied to either unique sequences or clonotypes. When computing clonality scores for unique sequences, we used sequence reads or template counts as frequencies. In the case of clonotypes, one can use either aggregate template counts for all sequences in the clonotype or member counts as the corresponding frequencies. All of the results are provided in Tables S1 and S2 in Supplementary Material.

### Calculating Ig Properties

#### Clonotype Ig Properties

While certain Ig properties, IGHV-family usage or CDR3 length, are constant for all members of the clonotype family (clonotype-invariable), others, such as SHM percentage or biophysical characteristics, vary across the clonotype family (clonotype-variable). We computed clonotype-weighted Ig properties for all clonotype-variant properties by using in-house developed Perl script with BioPerl modules (58). Specifically, we computed the aliphaticity, aromaticity, hydropathicity (gravy index), molecular weight, isoelectric pH, and electric charge at pH values of 5, 6, and 7 for the CDR3 loop of the IGH. We modified the electric charge calculation to reflect the fact that the CDR3 loop is not a free peptide but an element of the Ig protein chain. In addition, we calculated clonotype-weighted template counts and the percentage of SHMs in the available IGHV-gene segment. The latter property relies on the quality of the IMGT annotation (53, 54).

We performed clonotype weighting by using template counts of the sequences within each clonotype to remove the effect of primer bias and reduce contributions of inevitable PCR amplification and sequencing errors by scaling individual sequence contributions according to template counts normalized within their clonotypes (and not across the whole repertoire). This also allows pooling of data from multiple subjects, even when the total number of sequence reads varies among subjects. Importantly, this clonoype-based approach should work equally well with genomic DNA sequencing (where a single sequence read corresponds to a single B cell under the assumption of unbiased PCR amplification) and cDNA sequencing (where correspondence between the sequence reads and cells is less straightforward) (16).

#### Binning Repertoire Ig Properties

Because our statistical analysis tools were originally developed for discrete distributions (41, 42), we could directly apply them to distributions of nominal or discrete interval scale Ig properties, such as IGHV-family usage or CDR3 length, respectively. To carry out the analysis for continuous interval scale properties or for clonotype-weighted properties, we first converted the original dataset to a histogram with bins of finite size, where each data point was discretized by bin assignment and analyzed accordingly. In a sense, this technique generalizes the original KMS and SK methods to continuous variables by using binning. For continuous properties, we chose the bin size and bin centers to best reflect the range of values and shape of the continuous distribution. The bin size and bin centers were identical across all studied repertoires to allow direct comparisons.

Defining the bins for continuous properties is non-trivial and can have substantial effects on repertoire analysis. The influence of the bin size on the analysis is akin to quantiles. In the trivial case, where AC are in one, all-encompassing bin, comparisons yield no difference. If the bin size is too small, such that each clonotype occupies its own bin, no comparison is possible owing to lack of bin overlap between repertoires or too few data points within bins. In practice, we selected the bin size so that the resulting histogram captures the shape of the corresponding distributions.

### Statistical Analysis

#### Group, Individual, and Reduced Clonotype Repertoires

Although we carried out immunosequencing on each subject individually, we performed a group-level analysis by pooling the sequencing data within groups to provide better sampling and reduce the effect of individual variation on the observed trends. The repertoire size varied widely across subjects. When combining data from different subjects, we did not down-sample each repertoire to the smallest of the subjects (57). Importantly, because the independence assumption was satisfied at the clonotype level within each individual, the assumption remained valid when we pooled the clonotypes from multiple individuals into a single pooled repertoire.

To gain additional insight into the influence of subject- vs. group-level comparisons and the effect of sequence sample size within our repertoire analysis, we used two approaches to test the sensitivity of our results with respect to the number of subjects and clonotypes. First, we explored how each individual affected the group-level analysis by performing leave-one-out analyses for each subject of each group, i.e., removing the clonotype repertoire of one subject from the pooled repertoire of the corresponding group and repeating the analyses for each subject and each group. Second, we down-sampled the pooled clonotype repertoires and carried out comparisons between the unvaccinated and vaccinated conditions to determine the repertoire size at which significant differences between repertoires were no longer reliably identified.

#### Partitioning Clonotype Repertoires

To glean additional information about affinity maturation, we partitioned clonotypes by the number of members they contained. Clonotypes with only a single member are likely to represent direct, early-stage, descendants of naïve B cells, whereas clonotypes with many members may represent later-stage B cells that have actively undergone further rounds of affinity maturation. We performed statistical analysis by comparing repertoires for three different clonotype classes: all clonotypes (AC); branched clonotypes (BC), which had more than one member clone; and solitary clonotypes (SC), which contained only a single clone. (By definition, BC and SC are subsets of AC.)

#### Identifying Statistically Significant Differences within the Distributions

Both the SK and KMS methods provide critical *p*-values and estimated *p*-values for each proportion, or bin of the histogram. To determine whether a particular proportion was statistically significant, instead of using the Hochberg approach for multiple comparisons (59, 60), we used the stricter Bonferroni approach to minimize the number of false positives and to allow pairwise comparisons of more than two groups.

The Bonferroni approach requires the estimated *p*-value to be lower than its corresponding critical *p*-value. We used *p* = 0.05 to derive the critical *p_i_*-values for a bin of rank *i* as *p_i_* = *p*/*i*, where the integer rank is determined by ordering all of the bins in descending order of estimated *p*-values and numbering them consecutively starting with 1 and ending with *K*, the total number of bins. In addition to *p*-values, the SK method provides confidence intervals, which are essential for evaluating the size of the effect. We defined a difference between two distributions at a particular bin *i* as statistically significant if and only if the estimated *pi*-value was less than corresponding critical *pi*-value for *both* methods. This consensus approach ensured that any statistically significant differences identified were robust and of high confidence.

We ran in-house Perl software on a workstation to apply the SK and KMS methods as implemented in the WRS package (61) for the R statistical package (62). The user provided the pair of datasets as well as the Ig property to be compared, along with the bin size and the range of property values used to center the bins, as specified above. As inputs to the statistical analysis, the script used data points corresponding to clonotypes, whose values were either obtained directly from each clonotype (for discrete interval properties) or from the corresponding histograms (for continuous interval properties). In the latter case, each clonotype property value was set to the value of the center of the bin containing the original clonotype-weighted property value of the corresponding clonotype. The software then computed the difference for each proportion or histogram bin for the two datasets and the corresponding critical and estimated *p*-values. For each comparison, we defined a particular proportion or histogram bin as being significantly different between two distributions if the estimated *p*-values were below the corresponding critical *p*-values in both SK and KMS tests. We used gnuplot (63) to visualize the distributions and highlight statistically significant differences.

Finally, we demonstrated the advantage of the combined SK and KMS methods over standard methods by performing *t*-tests on V-SHM and CDR3 length data. We note that the *t*-test can only test *whether*, but not *where*, two distributions significantly differ. Furthermore, it cannot be applied to nominal scale properties, such as IGHV-family usage. We also applied a *t*-test-like *z*-test with Bonferroni correction to the proportions of IGHV-family usage (as described in Supplementary Material) and identified significant differences between the unvaccinated and vaccinated groups. However, we stress that the *z*-test assumes that the proportions comprising the corresponding distributions are normally distributed, whereas the combination of SK and KMS methods does not. This makes the latter potentially better suited for the irregular distributions often seen in repertoire data. Furthermore, to apply the *z*-test, the bins of interest should contain at least 10 clonotypes, in order to satisfy the success–failure requirement. An additional advantage of the SK and KMS methods is that they do not have such a requirement and were specifically developed to handle small samples.

#### Repertoire Size Constraints

For practical reasons, the tools we employed required that each repertoire contain no more than 10^5^ clonotypes (42). The maximum repertoire size that could be used for analysis varied between the two methods. The SK method, which is computationally more intensive, had a practical repertoire size limit of 10^5^ clonotypes. By contrast, the KMS method, which is rapid and analytical, could be applied to much larger datasets. In this work, we were able to use both methods.

## Results

### Immunosequencing

**Figure 2:**
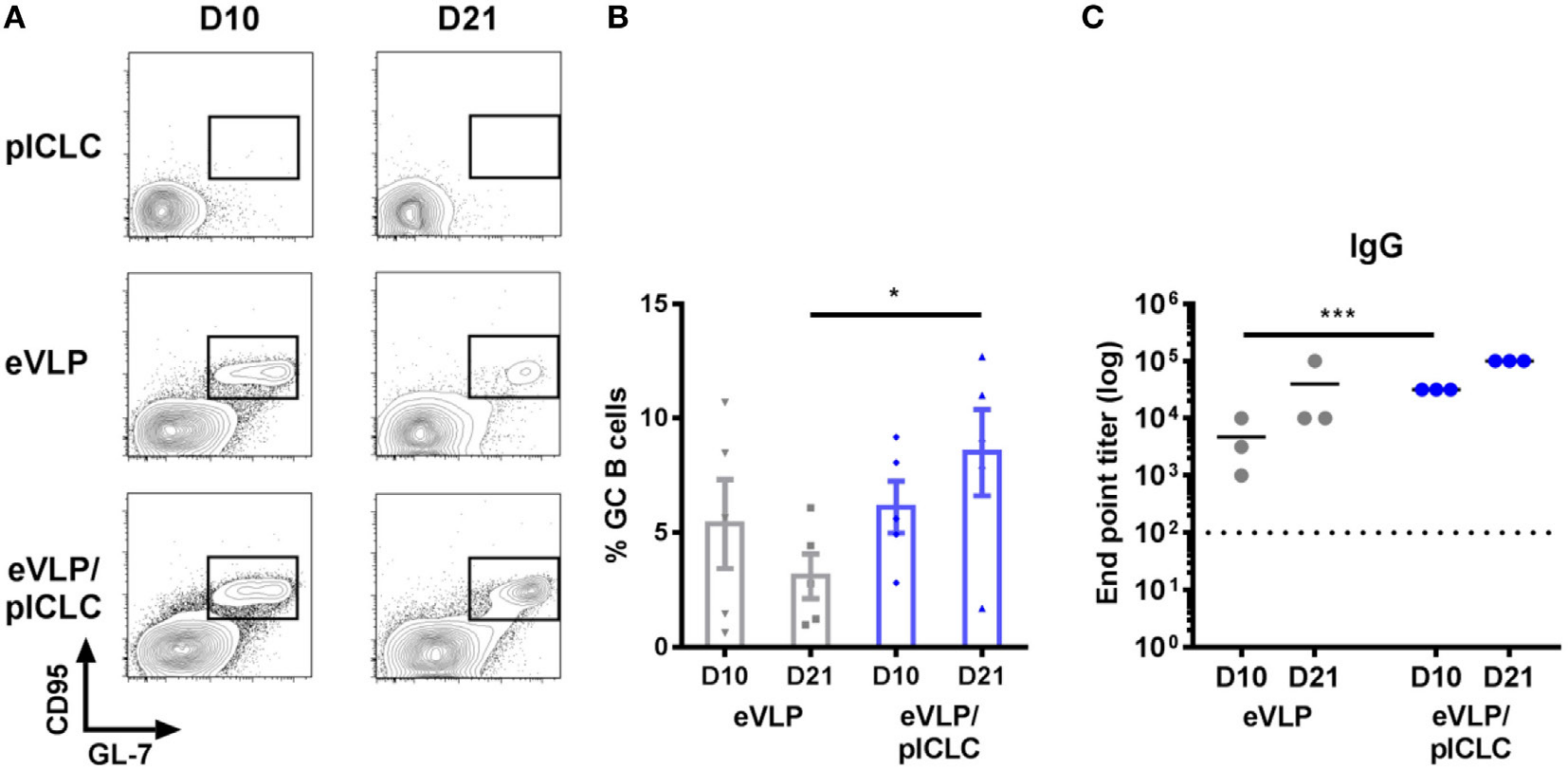
Ebola virus-like particle (eVLP)-mediated germinal center (GC) reactions following a single IM vaccination. Mice were vaccinated (intramuscularly *via* the caudal muscle of the right hind leg) with either eVLP alone or eVLP plus poly-ICLC (pICLC), and draining lymph nodes were isolated at the indicated time points. Single-cell suspensions were stained with B220, IgD, IgM, CD38, CD95, GL-7, and live/dead dye, after which they were collected by FACS. **(A)** Representative FACS plots of day 10 (D10) and D21 B220^+^CD95^+^GL7^+^ GC B cells. **(B)** Relative frequency following vaccination; error bars = SEM; *p < 0.05; *n* = 5 per group. **(C)** Serum samples were collected at the indicated time points and IgG responses against EBOV-GP_1,2_ following vaccination were measured by enzyme-linked immunosorbent assay. Endpoint titers were determined at a serum dilution corresponding to +0.2 SD from control OD readings (***p < 0.001; *n* = 3 per group).

Although previous eVLP studies in mice have focused on prime-boost vaccination schedules, we characterized GC B cells following single-dose vaccinations to exclude the effect of antigen-specific B-cell recall. Animals that received a single vaccination (intramuscularly through the caudal thigh) with eVLP, either alone or in combination with the pICLC adjuvant (eVLP/pICLC), exhibited robust GC B-cell reactions within the dLN, which were detectable as early as D10 following vaccination. However, inclusion of pICLC resulted in heightened D10 dLN cellularity (data not shown) and sustained GC formation on D21—a time point typically used for murine eVLP boosting (Figures 2A,B). Consistent with previous studies, eVLP-vaccinated mice displayed high levels of anti-EBOV GP_1,2_ titers, with an early increase of antibody responses in the presence of pICLC adjuvant (Figure 2C).

We focused on D10 eVLP-mediated responses, which allowed us to characterize the early vaccine GC B-cell seeding events and to increase the overall sequencing breadth for our repertoire analysis owing to the higher absolute GC B-cell cellularity compared to D21. Sorting of highly purified D10 GC B cells (B220^+^CD95^+^GL7^+^) resulted in the capture of 17,672 early GC B-cell sequences from the two primary vaccination groups [eVLP = 8,481 (*n* = 3); eVLP/pICLC = 9,191 (*n* = 3)], encompassing both productive and non-productive IGH rearrangements (Table S1 in Supplementary Material). Exclusion of out-of-frame reads, in-frame VDDJ reads, and reads with stop codons resulted in 12,163 (eVLP = 5,917; eVLP/pICLC = 6,246) total in-frame VDJ and VJ sequences without stop codons representing GC B-cell clones (Table 1; Table S1 in Supplementary Material). Removal of identical sequences derived from clonal expansion of a single B-cell clone identified 7,097 unique GC B-cell clones (eVLP = 3,516; eVLP/pICLC = 3,581). To establish a baseline repertoire, we additionally sorted naïve B cells (B220^+^IgM^+^IgD^+^GL7^−^CD95^−^) from unvaccinated control mice (*n* = 2) and identified a total of 35,509 unique in-frame VDJ and VJ sequences without stop codons (Table 1; Table S1 in Supplementary Material).

**Table 1:** B-cell receptor sequences.

Condition	Sample	# of cells	# of clones	Clonality	# of clonotypes	# of branched clonotypes
Naïve	Subject 1	24,885	17,938	0.01	16,031	1,186
	Subject 2	23,697	17,571	0.01	15,786	1,087
	Pooled	48,582	35,509	0.01	31,817	2,273
Ebola virus-like particle (eVLP)	Subject 3	4,274	2,451	0.11	908	310
	Subject 4	738	519	0.06	301	79
	Subject 5	905	546	0.12	201	72
	Pooled	5,917	3,516	0.10	1,410	461
eVLP/poly-ICLC	Subject 6	3,329	1,758	0.13	620	185
	Subject 7	2,623	1,590	0.12	561	185
	Subject 8	294	233	0.10	147	22
	Pooled	6,246	3,581	0.11	1,328	392

Following clonotype clustering of the sequence data, naïve B cells from the unvaccinated control group consisted of 31,817 total clonotypes (AC), whereas the eVLP and eVLP/pICLC groups netted 1,410 and 1,328 AC clonotypes, respectively (Table 1; Table S1 in Supplementary Material). Overall, ~30% of the clonotypes in both the eVLP and eVLP/pICLC conditions were BC, compared to less than 7% in naïve B-cell clonotypes of the control group.

### Clonality Scores of Repertoires

To define the overall clonal breadth of early GC B-cell responses following eVLP vaccination, we computed clonality scores, which evaluated whether the repertoire was equally represented by all clonotypes (score of 0) or dominated by a single clonotype (score of 1). The clonality scores reported in Table 1 were computed using clones with template counts as frequencies. Naïve B-cell clones displayed a near uniform distribution of clonotypes, both at the clonal level and after clonotype clustering (Table 1). The clonality scores of vaccine-induced GC B-cell repertoires for both eVLP and eVLP/pICLC groups were significantly higher than those of the naïve B-cell repertoire from the unvaccinated group (*p* < 0.05 and *p* < 0.01, respectively, based on Student’s *t*-test), suggesting an altered distribution of clonotypes due to clonal expansion.

### Calculating Ig Properties

The Ig properties calculated for each clone in the dataset are summarized in Table 2. These properties range in type, from nominal scale properties, such as IGHV-family usage, to discrete interval scale properties, such as CDR3 length, to continuous interval scale properties, such as V-SHM percentage. We also calculated a set of biophysical properties for the CDR3 amino acid sequence. Finally, because we performed the statistical analysis at the clonotype level, the properties were weight-averaged for each clonotype (see Materials and Methods). Certain properties, such as IGHV gene or CDR3 length, were invariable across all members of a clonotype. Other properties, such as the SHM percentage or biophysical characteristics, could vary between members of the clonotype as a result of the accumulation of mutations during affinity maturation; as such, these properties were variable.

**Table 2:** Ig Properties.

Property	Property type	Within clonotype
**Sequence properties**		
V gene	Nominal	Invariable
D gene	Nominal	Invariable
J gene	Nominal	Invariable
Complementarity-determining region (CDR3) length	Discrete	Invariable
V-SHM percentage	Continuous	Variable
**Biophysical CDR3 properties**		
Aliphaticity	Continuous	Variable
Aromaticity	Continuous	Variable
Hydropathicity	Continuous	Variable
Molecular weight	Continuous	Variable
Isoelectric point	Continuous	Variable
Electric charge (pH 5, 6, 7)	Continuous	Variable

### IGHV-Family Usage

#### IGHV-Family Usage in the Unvaccinated Group

**Figure 3:**
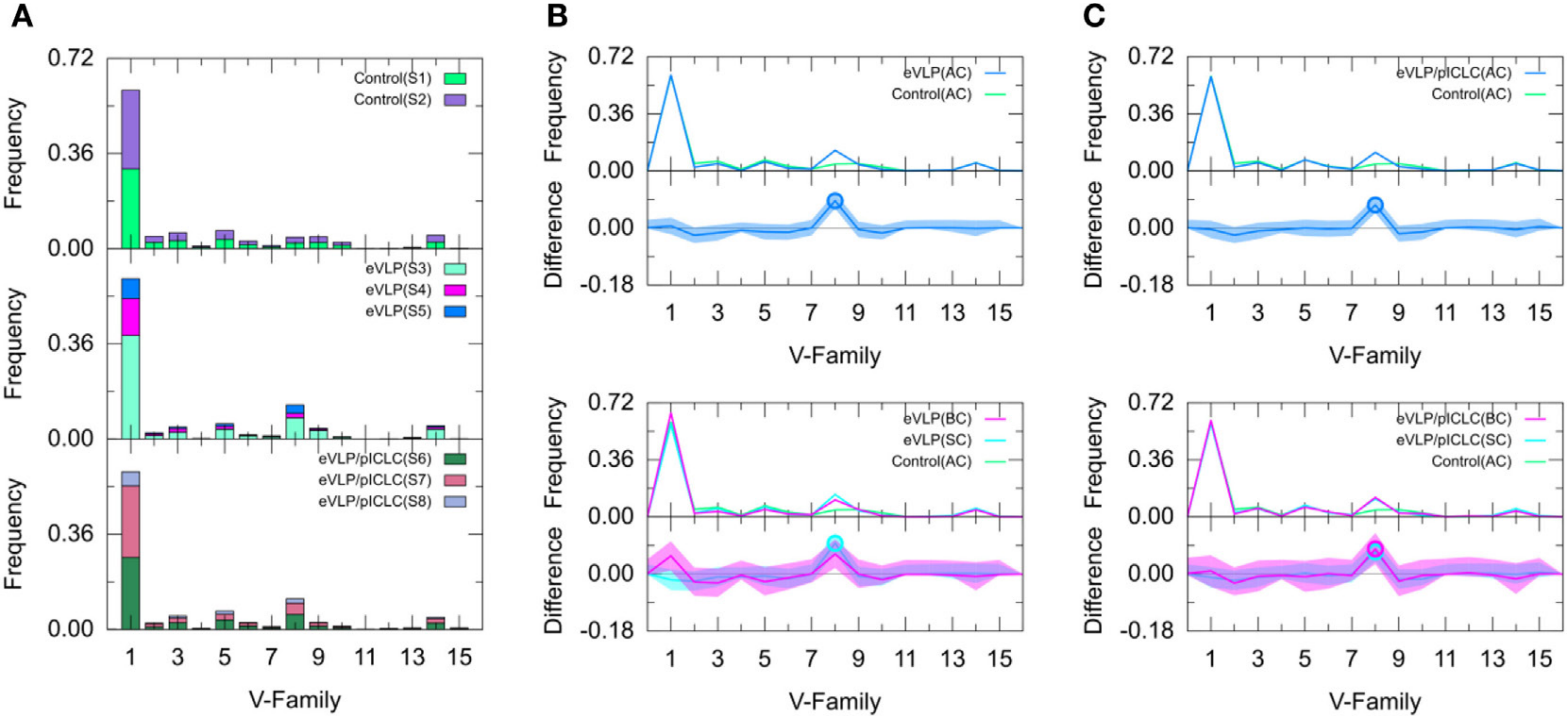
Clonotype-invariant Ig heavy chain variable-family usage. **(A)** The stacked bar charts show individual subject contributions. Subjects are numbered S1 through S8 in decreasing order of clonotype number within each group. Comparison of **(B)** Ebola virus-like particle (eVLP) and **(C)** eVLP/poly-ICLC (pICLC) groups with control group. Statistically significant differences are shown in the difference plots of **(B,C)** with open circles. The shaded areas around the main solid curves show confidence intervals.

Naïve B cells from the unvaccinated control group were used to establish baseline IGHV-family usage. The control repertoire was associated with clonotypes of IGHV families 1, 2, 3, 5, 8, 9, and 14 (Figure 3). The IGHV1 family dominated the control repertoire, representing 60.0% of the repertoire. The remaining clonotypes combined for 31.6% of the total control repertoire, with IGHV5 (6.9%) representing the second most frequent subgroup (Figure 3). These numbers were similar for both subjects of the unvaccinated control group.

#### Comparison of IGHV-Family Usage in Vaccinated and Unvaccinated Groups

We compared the IGHV-family usage in GC B-cell repertoires from the eVLP- and eVLP/pICLC-immunized mice with the naïve B-cell repertoire from unvaccinated control mice. The IGHV1 family was the most frequent in both vaccinated groups, representing 60.6 and 59.6% for eVLP and eVLP/pICLC, respectively (Figure 3). By contrast, the proportion of the IGHV8-family clonotypes was statistically significantly higher in both the eVLP and eVLP/pICLC conditions (12.9 and 11.6% of clonotypes, respectively; by consensus *p*-values from SK and KMS methods), compared to the repertoire from the control group. The proportion of the IGHV8-family clonotypes in the rearranged genes in response to eVLP and eVLP/pICLC was the second largest across all subjects within each group (Figure 3A).

We applied a *z*-test with Bonferroni correction to the AC repertoires and confirmed that the proportion of the IGHV8-family clonotypes statistically significantly increased in both vaccinated groups with respect to that of the control group. In addition, relative to the control group, the *z*-test revealed significantly reduced proportions of IGHV2, IGHV4, IGHV6, and IGHV10 for the eVLP group and IGHV2, IGHV9, and IGHV10 for the eVLP/pICLC group.

Comparing the SC and BC repertoires from immunized mice separately with the naïve B-cell repertoire from unvaccinated mice, we found statistically significantly increased IGHV8-family usage in the SC repertoires of both eVLP and eVLP/pICLC conditions (by consensus of SK and KMS methods in both cases), as well as in the BC repertoire of the eVLP/pICLC condition. A notable increase in IGHV1-family usage was observed in the BC repertoire of the eVLP group, which may have resulted in the dilution or loss of statistical significance for the IGHV8-family subgroup in the BC repertoire in this condition.

### SHMs in IGHV Segment (V-SHM)

**Figure 4:**
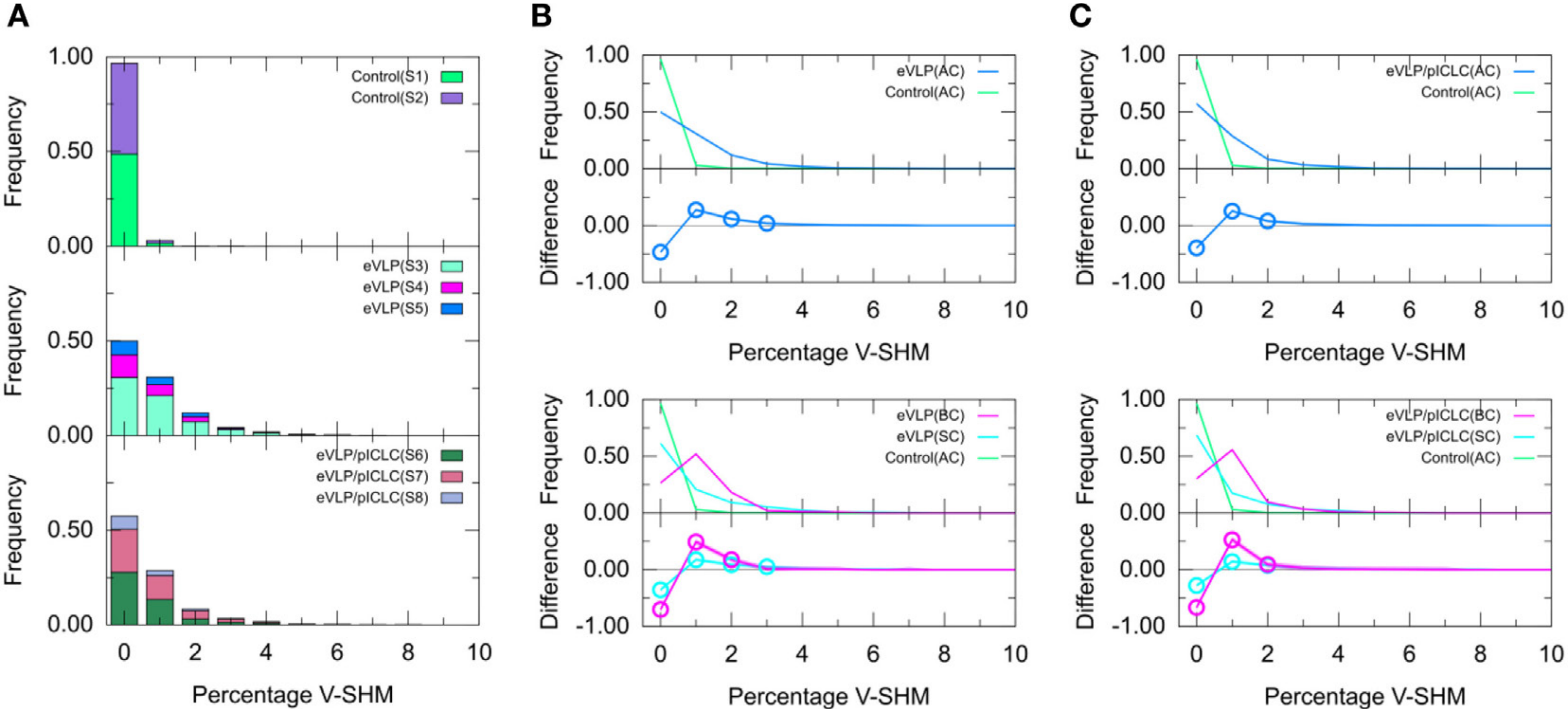
Clonotype-weighted SHM percentage in Ig heavy chain variable-gene segment (V-SHM). **(A)** The stacked bar charts show individual subject contributions. Subjects are numbered S1 through S8 in decreasing order of clonotype number within each group. Comparison of **(B)** Ebola virus-like particle (eVLP) and **(C)** eVLP/poly-ICLC (pICLC) groups with the control group. Statistically significant differences are shown in the difference plots of **(B,C)** with open circles. Although barely noticeable, the shaded areas around the main solid curves show confidence intervals.

We next compared the clonotype-weighted percentage of SHMs in the IGHV gene segment between unvaccinated control mice and eVLP-vaccinated mice. Most clonotypes from the naïve B-cell repertoire of the unvaccinated control group were completely devoid of detectable V-SHMs and only a small proportion (3.0%) showed a V-SHM percentage of 1% (Figure 4A).

By sharp contrast, GC B-cell repertoires from either immunization condition showed a much smaller proportion of clonotypes without V-SHMs and greater proportions of clonotypes with V-SHM percentages of 1, 2, and 3% (Figure 4A). In particular, the repertoire of eVLP-immunized mice was significantly different from that of the control group at V-SHM percentages of 0, 1, 2, and 3% (Figure 4B). The repertoire from eVLP/pICLC-immunized mice was significantly different at V-SHM percentages of 0, 1, and 2% (Figure 4C). Hence, the repertoire of eVLP-immunized mice was slightly more mutated. The mean V-SHM values were 0.04, 0.08, and 0.07% for the control, eVLP, and eVLP/pICLC groups, respectively. Applying a standard *t*-test to these values revealed that the vaccinated groups were significantly different from the control group (*p* < 0.05). However, we note that V-SHM is not normally distributed and, as such, the mean V-SHM is a poor surrogate for the overall distribution.

Partitioning of the clonotypes into SC and BC subsets provided additional information about their composition. Specifically, BC repertoires typically showed a much lower proportion of clonotypes with a V-SHM percentage of 0%, with the proportion peaking at 1% (Figure 4B,C, magenta line). By contrast, although SC repertoires showed a significantly lower proportion of clonotypes with a V-SHM percentage of 0% than the naïve B-cell AC repertoire of the unvaccinated control group, the proportion in vaccinated groups showed no peak and instead quickly decayed to near zero at a V-SHM percentage of 3% (Figure 4B,C, light blue line). Interestingly, the SC subset of eVLP-immunized mice contributed to the significant elevation in the proportion of clonotypes with a V-SHM percentage of 3% (Figure 4B, light blue open circle).

### CDR3 Properties

#### CDR3 Length

**Figure 5:**
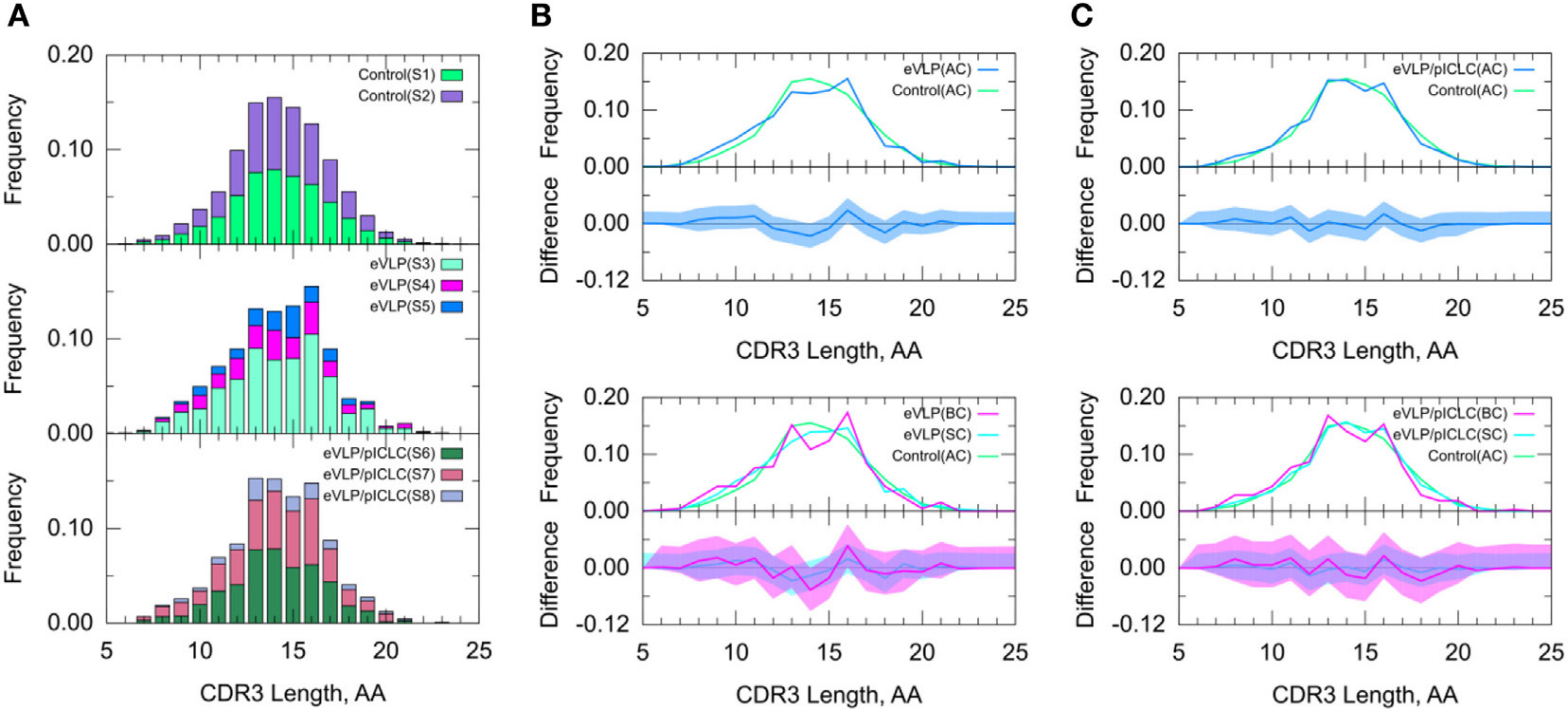
Clonotype-invariant complementarity-determining region (CDR3) length. **(A)** The stacked bar charts show individual subject contributions. Subjects are numbered S1 through S3 in decreasing order of clonotype number within each group. Comparison of **(B)** Ebola virus-like particle (eVLP) and **(C)** eVLP/poly-ICLC (pICLC) groups with the control group. The shaded areas around the main solid curves show confidence intervals. See text for definitions of all clonotypes (AC), branched clonotypes (BC), and solitary clonotypes (SC) repertoire subsets.

Because the CDR3 loop is a critical determinant of antigen recognition, we next compared the CDR3 lengths of sequenced B-cell clones. The B-cell repertoire from the unvaccinated control group displayed a near-normal distribution of CDR3 lengths. By contrast, both eVLP- and eVLP/pICLC-immunized mice showed larger proportions of clonotypes with CDR3 lengths of 13 and 16 amino acids (Figure 5), although the trend was not significant. These results were corroborated by a standard *t*-test on the mean CDR3 lengths. Decomposition of AC repertoires into SC and BC repertoires also showed enrichment of CDR3 lengths 13 and 16, particularly among BC repertoires, but again this trend was not significant (Figures 5B,C).

A standard *t*-test may be more appropriate for CDR3 length than for V-SHM, because unlike the latter, CDR3 length appears to be approximately normally distributed across repertoires. The results of the *t*-test agreed with those of the KMS and SK tests—there was no statistically significant difference in the CDR3 length distribution between the control and vaccinated conditions.

#### Biophysical Indices

We performed statistical analyses for clonotype-weighted CDR3 loop biophysical properties listed in Table 2 (refer to Figures S1–S8 in Supplementary Material). Both vaccinated groups showed a consistent shift toward an increased aliphatic index compared to that of the unvaccinated control group (Figure S6 in Supplementary Material). Both vaccinated groups showed a large decrease in the proportion of clonotypes compared to B-cell repertoires from the unvaccinated control group, with a CDR3 aliphaticity score of 10; this difference was statistically significant for the eVLP/pICLC condition (Figure S6B in Supplementary Material). We also identified notable differences in CDR3 aromatic amino acid usage between the three groups. While the eVLP/pICLC group showed a similar aromaticity profile to the B-cell repertoire of the control group, the eVLP group differed from both the control and eVLP/pICLC groups. The eVLP group showed a significant decrease in the proportion of clonotypes, with a CDR3 loop aromaticity value of 30 compared to both control and eVLP/pICLC repertoires, and a concomitant increase in the proportion of clonotypes with an aromaticity value of 20 relative to the eVLP/pICLC group (Figures S5A,C in Supplementary Material). Analysis of the remaining biophysical properties did not reveal any significant differences between the groups.

### Effect of pICLC Adjuvant

**Figure 6:**
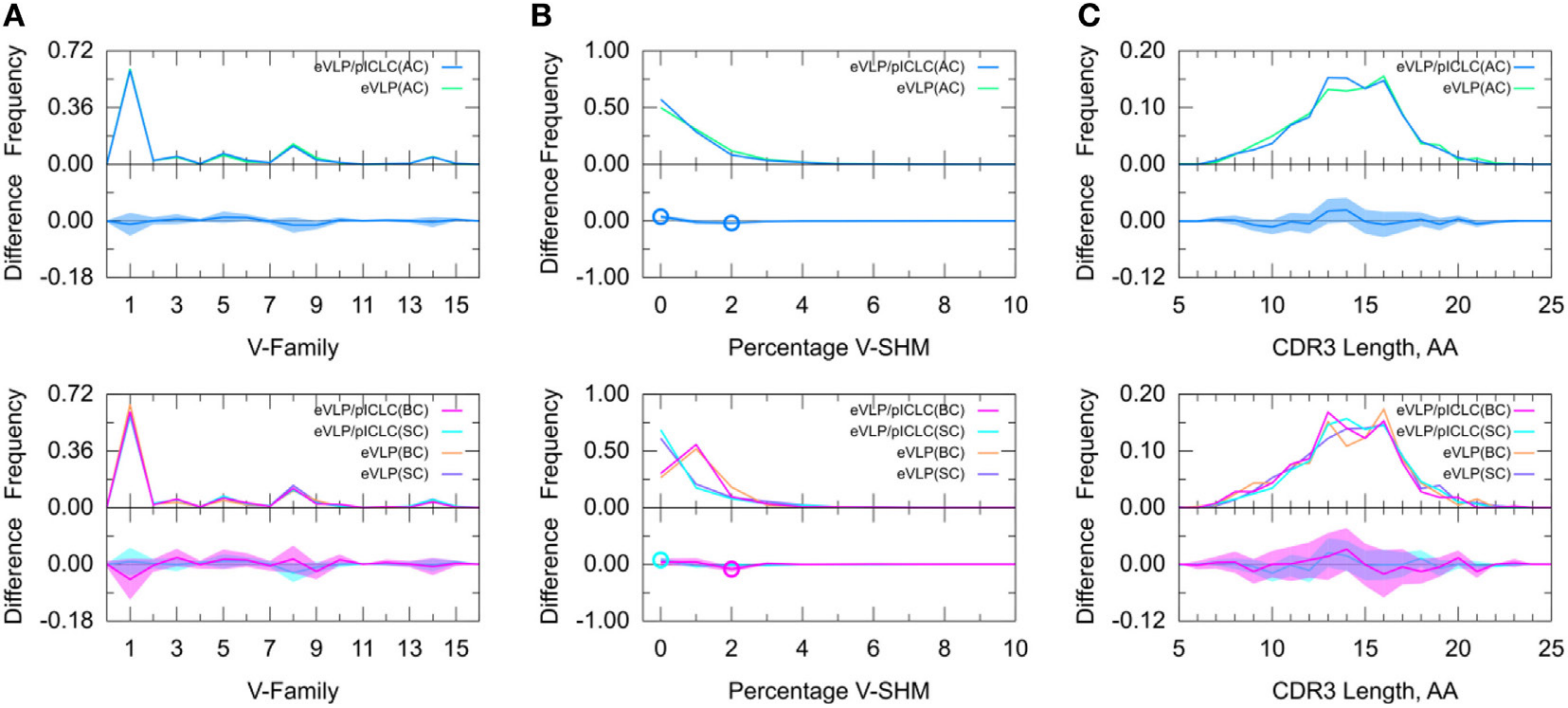
Direct comparison of Ebola virus-like particle (eVLP) and eVLP/poly-ICLC (pICLC groups). **(A)** Clonotype-invariant Ig heavy chain variable-family usage. **(B)** Clonotype-weighted SHM percentage in V-gene segment (V-SHM). **(C)** Clonotype-invariant complementarity-determining region (CDR3) length. Open circles indicate statistically significant differences in the difference plots. The shaded areas around the main solid curves show confidence intervals.

To further determine adjuvant effects on the GC B-cell repertoire, we directly compared the two vaccinated groups. The results of this inter-group comparison (Figure 6) were largely consistent with an indirect comparison of these groups *via* comparisons with the naïve B-cell repertoire from the unvaccinated control group, in that there were no major differences between the two vaccine conditions. However, the distribution of V-SHM differed slightly between the vaccine conditions. Specifically, in the absence of the adjuvant, we found a modest but statistically significant increase in the proportion of clonotypes without mutations and a similar decrease in the proportion of clonotypes with 2% V-SHM. This is consistent with our finding that GC B-cell repertoires were slightly more mutated in the eVLP group than in the eVLP/pICLC group. This difference was also observed with a *t*-test comparison of the mean V-SHM values.

Decomposition of the repertoire into SC or BC revealed that the increase in the proportion of clonotypes with 0% V-SHM in the eVLP/pICLC group was associated with SC, whereas the decrease in the proportion of clonotypes with 2% V-SHM was associated with BC. Overall, these results suggest that differences in the repertoires between the vaccine conditions are minimal at the early stages of the GC response, but that in the non-adjuvant condition, there may be a slightly larger proportion of clonotypes undergoing clonal expansion (Table S1 in Supplementary Material), as reflected in the slightly higher V-SHM.

### Assessing Robustness of Observed Repertoire Features

IGHV8-family usage was significantly enriched for eVLP-mediated GC B-cell clonotype repertoires. To test the robustness of this repertoire signature, we performed additional tests by down-sampling the AC repertoires in several ways (Table S3 in Supplementary Material) to test how sensitive these results were to varying sample sizes. For each target repertoire size, we drew 10 independent samples from the corresponding complete repertoires and performed 10 corresponding statistical comparisons. We report the results of the comparisons as percentage outcomes based on these 10 independent comparisons for each sample size.

First, we down-sampled the naïve B-cell repertoire of the control group from 100% (*n* = 31,817) down to 25, 10, 5, and 1%, while leaving the repertoire size of the vaccinated groups unchanged. Down-sampling to 5% repertoire size in the control group revealed that IGHV8 family remained statistically significantly enriched in the vaccinated groups; however, in down-sampling to 1% of the control group, this finding remained significant in only 60% of the comparisons (Table S3 in Supplementary Material).

Second, we down-sampled the GC B-cell repertoires from the vaccinated groups from 100% [*n* = 1,410 (eVLP) and *n* = 1,328 (eVLP/pICLC)] down to 50 and 25%, and compared these samples with 100% of the control group repertoire. IGHV8-family usage was still significantly enriched in the vaccinated condition in 90–100% of the comparisons after 50% down-sampling of the B-cell repertoires from the vaccinated groups (Table S3 in Supplementary Material). At down-sampling to 25% of the repertoire size in the vaccinated groups, this finding was observed in only 40–50% of the comparisons. Based on this finding, we estimated that a clonotype repertoire size of around 700 is needed to reliably detect the increase in IGHV8-family usage in vaccinated conditions with statistical significance.

Since our repertoire from the control group came from naïve B cells, whereas our repertoires from the vaccinated groups came from GC B cells, there was a significant size imbalance between the control and vaccinated group repertoires—the control group repertoire was ~20 times larger than the vaccinated group repertoires. To assess the effect of sample size imbalance on the outcome of comparisons between repertoires, we down-sampled both control and vaccinated repertoires to an equal number of clonotypes at 1,200, 800, and 400. At balanced repertoire sizes of 1,200 and 800, the IGHV8-family usage was enhanced in the vaccinated conditions in 100% of the comparisons; at repertoire sizes of 400, this finding was observed in at least 80% of the comparisons (Table S3 in Supplementary Material). This suggests that the large relative size of the control group repertoire did not have a major effect on the repertoire comparisons and that a minimum repertoire size of ~700 clonotypes is the primary limiting factor in the analysis.

Finally, we performed a leave-one-out test to assess the effect of pooling individual subjects in the group, by leaving the AC repertoire of one subject out of the group, repeating this for all subjects in the eVLP and eVLP/pICLC groups. Leaving a subject out of the vaccinated group altered the result in only one case. Specifically, leaving subject S3 out of the eVLP group resulted in a loss of statistical significance for the IGHV8-family enrichment. Note, however, that subject S3 had 908 of the 1,410 clonotypes of the eVLP group. Leaving S3 out results in a repertoire of only 502 clonotypes, which is below our estimate of the 700 required for detection of the IGHV8-family enrichment.

## Discussion

The exquisite diversity of humoral responses is established from a stochastic pool of naïve B-cell progenitors recruited into GC reactions following antigen recognition. Seeding of these early GC B cells provides the initial framework for successive rounds of inter- and intra-clonal competition for both T-cell help and antigen within secondary lymphoid tissue. The resultant pool of antigen-specific B cells selected through Darwinian-like processes may provide both immediate (e.g., plasma B cells) and life-long (e.g., memory B cells) protection against pathogen exposure. However, the following issues largely remain unresolved for any given antigen: (1) the frequency of precursor antigen-specific naïve B cells, (2) the clonal diversity of early GC B-cell seeding events, and (3) how GC B-cell repertoires evolve during the antigen selection process. Recent studies have suggested that unlike simple model antigens, such as haptens, which typically induce monoclonal or oligoclonal B-cell responses, complex vaccine antigens, such as viruses, bacteria, or recombinant proteins, induce highly polyclonal responses at both early and late stages of antigen selection.

Previous studies have demonstrated that vaccination with eVLP, a complex virus-like particle antigen that closely resembles EBOV, results in strong humoral responses and protective immunity against an otherwise lethal dose of EBOV in mice (47, 48). More recently, we have shown that inclusion of the dsRNA adjuvant, pICLC, during eVLP vaccination augmented anti-EBOV GP_1,2_ responses, enhanced GC B-cell formation, and provided increased efficacy in mice (50, 51). Here, we build upon these findings and provide evidence that inclusion of pICLC alters eVLP-mediated GC B-cell dynamics resulting in a significantly sustained GC responses for at least 3 weeks after a single-dose vaccination. These findings may be informative for controlling GC B-cell output through refinement of vaccine scheduling (e.g., altering timing for additional vaccine boosting).

To provide further insights into eVLP-mediated B-cell responses, we used repertoire-scale immunosequencing in conjunction with a novel statistical approach to characterize the clonal diversity of the early GC B-cell response to eVLP and quantitatively compare GC B-cell repertoires under different immunization conditions. We partitioned the resulting clonotype repertoires into SC and BC repertoires, which may, respectively, reflect early-stage direct descendants of naïve B cells and later-stage more distant descendants that have undergone successive rounds of affinity maturation. By using this approach, we compared pooled repertoires from mice in unimmunized, eVLP-immunized, and eVLP/pICLC-immunized conditions to characterize the early GC B-cell repertoire in eVLP-immunized mice and to determine whether the addition of the pICLC adjuvant alters the early-stage GC B-cell response.

Diversity measurements of BCR sequences from sorted GC B cells, such as the clonality scores revealed that early eVLP-mediated GC B-cell responses were highly diverse. Consistent with GC selection, the clonality of GC B-cell repertoires in both eVLP and eVLP/pICLC groups increased nearly eightfold relative to the naïve B-cell repertoire from unvaccinated control group. Nonetheless, the GC repertoire in eVLP-immunized mice was still highly and equally diverse for both vaccination conditions. This diversity may reflect the status of the GC reaction at an early time point (D10), in agreement with a previous study (5). Furthermore, the lack of an early clonal convergence in GC B cells may suggest that anti-EBOV GP_1,2_ humoral responses at D10 may also be highly diverse.

Clonotype clustering also revealed a highly diverse eVLP GC B-cell response. In total, we identified over 7,000 GC B-cell clones accounting for almost 3,000 unique B-cell clonotypes in eVLP-immunized mice. Partitioning the repertoire into SC and BC repertoires showed that the number of templates per clonotype for the BC repertoire was higher than that for the AC repertoire, consistent with the theory that the BC repertoire reflects clonotypes that have undergone further rounds of affinity maturation. Interestingly, despite their similar genetic backgrounds, eVLP-vaccinated mice showed little overlap of GC B-cell clones or clonotypes (0.37% of unique B-cell clones shared by the two vaccinated groups; see Supplementary Material for details), underscoring the intrinsic diversity of the early GC B-cell response to eVLP vaccination.

In a repertoire-level comparison of the eVLP and eVLP/pICLC conditions with unvaccinated mice, we identified several statistically significant repertoire features unique to eVLP-immunized mice. In terms of V-family usage, we found that although IGHV1 was the predominant V-gene family used in all conditions, consistent with previous studies (11), and that IGHV8 family was statistically significantly enriched in both eVLP and eVLP/pICLC conditions, accounting for almost 12% of the repertoire. This enrichment was present in the SC repertoire, suggesting that it occurs even at an early-stage of the GC reaction. In the presence of the adjuvant, IGHV8-family enrichment was also found in the BC repertoire, suggesting that this effect may propagate through the affinity maturation process. Together, the enrichment of the IGHV8 family in the vaccinated groups can be considered a signature of the eVLP-mediated response. In line with these findings, a previous report on a much more limited repertoire independently identified IGHV8-family usage in response to a similar EBOV antigen within plasma B-cell populations (21). Although a statistical analysis of the plasma B-cell repertoire was not offered in that study, its findings reinforce the notion that IGHV8 GC B cells are likely selected to become functional antibody producing cells. Moreover, the similarity of repertoires suggests a common usage of IGHV8 B-cell clones despite differences in EBOV vaccination, use of adjuvant, or mouse strain.

We also identified significant differences in the percentage of V-SHMs between the vaccinated and unvaccinated groups. Naïve B-cell repertoires from the unvaccinated control group were nearly devoid of SHMs, whereas GC B-cell repertoires from both vaccinated groups displayed statistically significantly higher percentage of V-SHM. Vaccine-induced BC repertoires displayed the highest percentage of V-SHM, consistent with our interpretation that clonotypes in the BC repertoires may represent B cells that engaged in further rounds of antigen-specific affinity maturation. SC repertoires, by contrast, predominantly contained unmutated B-cell clones. Nevertheless, a small fraction of SC repertoires contained a non-zero percentage of V-SHMs. These SC repertoires may represent “orphaned” B-cell clones, i.e., lineages that evolved quickly but failed to improve affinity after several cycles of affinity maturation. It should also be noted that although our IGH sequences only provided coverage beginning from the FR3, we still identified substantial nucleotide mutations in the IGHV gene, as evidenced by the clonal expansion and affinity maturation within GC B cells. However, our V-SHM percentage likely underestimate the complete mutational load associated with more distal substitutions beginning within FR1 and across CDR1 and CDR2.

To determine the adjuvant effect on early-stage GC B-cell responses, we directly compared eVLP and eVLP/pICLC repertoires. We found modest but statistically significant differences in the V-SHM percentage between the eVLP and eVLP/pICLC groups. In the absence of the adjuvant, the eVLP-mediated GC responses appeared to have a slightly increased proportion of clonotypes undergoing clonal expansion with higher SHM percentage. In addition, we found more robust differences in CDR3 loop aromaticity, suggesting that eVLP- and eVLP/pICLC-induced GC B cells may target different vaccine epitopes. Nevertheless, we concluded that adjuvant inclusion had little if any effect on other early-stage GC B-cell repertoire properties, such as clonality, V-family usage, or other CDR3 properties.

Finally, we compared our novel statistical approach that combines clonotype clustering with the SK and KMS statistical methods to a more traditional *t*-test. Our repertoire analysis approach demonstrates that while such approaches are capable of determining *whether* two repertoires differ from each other, the SK and KMS methods can identify precisely *where* they differ and provide the magnitude of the observed differences *via* confidence intervals.

We further assessed the robustness of our results by carrying out a leave-one-out analysis to determine how individual repertoire variation affects results obtained from the pooled repertoires. Our results showed that, in most cases, the statistical differences found in the pooled repertoire were robust to individual repertoire variation and not the result of the contribution of any single mouse to the pool.

Also, we carried out down-sampling to determine how significant differences identified using our approach are affected by repertoire size. This in turn demonstrated that our repertoire analysis can be applied to comparisons of both balanced and unbalanced repertoires in terms of repertoire size and that the minimum repertoire size needed to reliably identify statistically significant differences in V-family usage using this approach is ~700 clonotypes.

Together, our findings suggest that early GC B-cell seeding events following vaccination with a complex EBOV antigen (eVLP) involves the recruitment of a diverse polyclonal pool of antigen-specific naïve progenitors. The combined sequences from all vaccinated mice identified close to 10^4^ unique GC B-cell clones associated with early eVLP vaccine responses. Despite the observed eVLP polyclonal response, we found that selection of IGHV8 family B cells was a robust signature associated with eVLP vaccination and that the addition of an adjuvant enhanced IGVH8 B-cell clonal expansion. The increase in IGHV8-family usage in immunized mouse repertoires likely represents the initial selection process that works on the GC seeding of naïve antigen-specific B cells. Intriguingly, this initial selection can be diluted and perhaps even lost during affinity maturation, as clonotypes from other IGHV families improve affinity toward the antigen. In this study, we observed the IGHV1 family gain ground as a result of affinity maturation. A recent study reported similar observations, using different complex antigens and analyzing B-cell repertoires from data at multiple time points (5). Hence, partitioning of the repertoire into SC and BC subsets captures some of the temporal characteristics of the GC reaction from a single, early GC time point.

## Conclusion

We developed a novel statistical approach for quantitatively comparing the Ig properties of immunosequenced BCR repertoires and determining not just *whether* but also *where* the Ig property distributions between two repertoires differ. We used the SK and KMS methods from Wilcox’ robust statistics toolbox, which, unlike most commonly used statistical approaches, do not make assumptions about the underlying Ig property distributions and can be used to determine the confidence intervals necessary to assess the size of the effects and their potential biological relevance. We applied this statistical approach to identify GC B-cell repertoire signatures associated with an Ebola vaccine candidate, and carried out further tests to determine the robustness of these observations with respect to repertoire size and subject level variation. We further partitioned the repertoire into SC and BC clonotypes to provide additional insight into the GC reaction, making it possible to capture the temporal characteristics of early GC B-cell reactions from a single time point. Our results revealed that eVLP repertoires showed significantly higher SHM rates than unvaccinated repertoires and that IGHV8-family usage was significantly enhanced in the eVLP-vaccinated conditions. Finally, our findings underscore the general applicability of this statistical approach for comparing BCR repertoires in a range of immunological studies, from autoimmune disorders, to cancer, to infection and vaccination.

## Ethics Statement

Animal work was performed under a protocol approved by the USAMRIID Institutional Animal Care and Use Committee, in compliance with the US Animal Welfare Act, Public Health Service Policy, and other federal statutes and regulations relating to animals and experiments involving animals. The facility where this research was conducted (USAMRIID) is accredited by the Association for Assessment and Accreditation of Laboratory Animal Care International, and adheres to the principles stated in the Guide for the Care and Use of Laboratory Animals (National Research Council, 2011).

## Author Contributions

Conception or design of the work: CC, SC, and IK. Data collection: CC, SS, and JB. Data analysis and interpretation: CC, SC, IK, and DL. Drafting of the article: IK. Critical revision of the article: CC, SC, AW, and SB. Final approval of the version to be published: CC, SC, IK, AW, SB, DL, SS, and JB.

## Conflict of Interest Statement

The authors declare that the research was conducted in the absence of any commercial or financial relationships that could be construed as a potential conflict of interest.
