# Supplementary material for: Quantitative Analysis of Repertoire-Scale Immunoglobulin Properties in Vaccine-Induced B-Cell Responses
Source: Front Immunol. 2017 Aug 14;8:910. doi: 10.3389/fimmu.2017.00910 (PMC5557726; doi:10.3389/fimmu.2017.00910)
Supplement: Supplementary file 1 [file Data_Sheet_1.DOCX]

Supplementary Material

Quantitative analysis of repertoire-scale immunoglobulin properties in vaccine-induced B-cell responses

Ilja V. Khavrutskii^1^, Sidhartha Chaudhury*^1^, Sabrina Stronsky^2^, Donald W. Lee^1^, Jacqueline Benko^2^, Anders Wallqvist^1^, Sina Bavari^2^, and Christopher L. Cooper*^2^

^1^Department of Defense Biotechnology High Performance Computing Software Applications Institute, Telemedicine and Advanced Technology Research Center, United States Army Medical Research and Materiel Command, Fort Detrick, Maryland, USA

^2^ Molecular and Translational Sciences, United States Army Medical Research Institute of Infectious Diseases (USAMRIID), Fort Detrick, Maryland, USA

**^*^Corresponding Authors:**
[sidhartha.chaudhury.civ@mail.mil](mailto:sidhartha.chaudhury.civ@mail.mil)

[christopher.l.cooper8.ctr@mail.mil](mailto:christopher.l.cooper8.ctr@mail.mil)

# Z-test with Bonferroni correction for multiple hypothesis testing

To find statistically significant differences between two groups of lymphocytes by examining the properties of their clonotype repertoires, we follow the procedure described below. For a given property of the clonotype repertoires of sizes *C1* and *C2*, we first build the corresponding distributions at the clonotype level, using clonotype counts and the corresponding clonotype-weighted values of the property. In the case of a continuous interval scale property, we build discrete histograms by binning the clonotype property values over the clonotype repertoires to ensure that the distributions of the property of interest from the two groups have the same number of bins, which are centered at the same values and of equal width. We try to choose the bin size so that the bins of interest have at least 10 clonotypes to satisfy the success-failure requirement of the z-test. We use the number of clonotypes that fall within each bin as the corresponding counts and do not normalize them. Bins with zero clonotype counts in one or both groups will be excluded from the analysis because they do not satisfy the success-failure requirement.

Suppose the two histograms of the property of interest to be compared each have *K* bins, where the bins are numbered *1* through *K* as they appear in each histogram. For a particular bin *i*, the clonotype counts in the corresponding histograms of the two groups are *c1,i* and *c2,i*. We then test *K* hypotheses that the proportions *p1,i* = *c1,i / C1* and *p2,i* = *c2,i / C2* are equal in both groups, using a corresponding binomial proportion z-test. Specifically, we compute the pooled probability of finding a clonotype in bin *i*, or the expected proportion of the clonotypes in bin *i*, as *p12,i* = *(c1,i + c2,i) / (C1 + C2)* and compute the pooled standard error *SE12,i* = sqrt [*p12,i* *** *(1 – p12,i) / C1* + *p12,i* *** *(1 – p12,i) / C2*]. The z-test statistic is given as *Z12,i* = *(p1,i – p2,i ) / SE12,i*. Tables to find the corresponding *p*-value *p12,i* are available. These *p12,i* values for *i = 1, …, K* bins are then listed in descending order and compared to the critical *p*-value *pB,k_i_* = *p / k_i_*, where *k_i_* is the corresponding order number of bin *i* according to its *p12,i*-value, *p* = 0.05, and *B* stands for Bonferroni. For bins where *p12,k_i_ < pB,k_i_*, we can consider the null hypothesis to be false and that the difference in bin *i* is significant.

# Shared CDR3 amino acid sequences

The percentage of group-level overlap S*_ij_* of shared sequences was computed as

$S_{ij}=\frac{n_{ij}}{\sqrt{N_{i}*N_{j}}}*100$,

where *n_ij_* is the number of shared productive sequences between groups *i* and *j*, and *N_i_* and *N_j_* are the total numbers of productive sequences in these two groups. Two sequences are considered shared by groups *i* and *j* if at least one CDR3 amino acid (AA) sequence is found in at least one subject of group *i* and at least one subject of group *j*. We also relaxed the criteria for matching sequences to allow for one AA mismatch. The numbers are summarized in the table below.

|  | | | | **Exact AA match** | | **Single AA mismatch** | |
| --- | --- | --- | --- | --- | --- | --- | --- |
| *i* | *j* | *N_i_* | *N_j_* | *n_ij_* | *S_ij_* | *n_ij_* | *Sij* |
| **eVLP/pICLC** | **eVLP** | 3,581 | 3,516 | 13 | 0.37 | 43 | 1.21 |
| **eVLP** | **Naïve** | 3,516 | 35,509 | 50 | 0.45 | 145 | 1.30 |
| **eVLP/pICLC** | **Naïve** | 3,581 | 35,509 | 41 | 0.36 | 128 | 1.45 |

# Shared sequences: Supplementary Figures and Tables

## Supplementary Figures


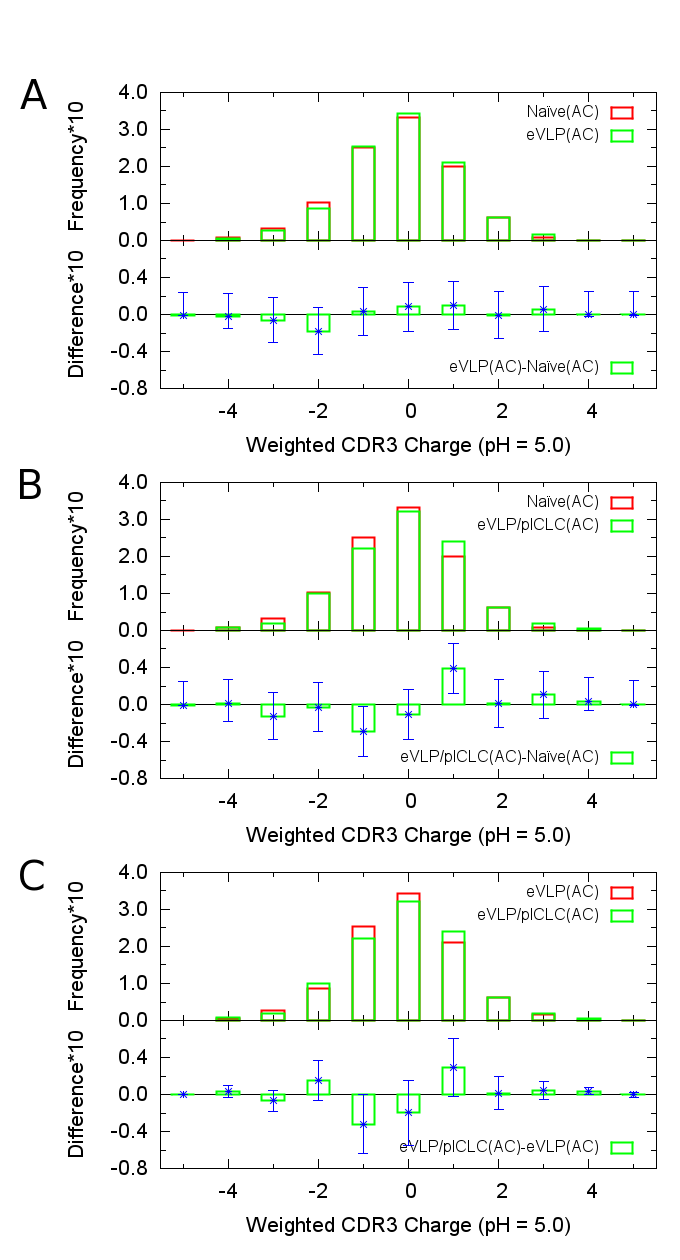


**Figure S1.** Pair-wise comparisons of distributions of clonotype-weighted charge of the CDR3 loop at pH = 5. A) eVLP group relative to Naïve group. B) eVLP/pICLC group relative to Naïve group. C) eVLP/pICLC group relative to eVLP group. All clonotypes (AC) were used in these comparisons. In each subplot, the top pane shows normalized histograms (bin size: 1 charge unit) representing the corresponding distributions; the bottom pane shows the differences between the corresponding histograms, along with confidence intervals, derived by using the Kulinskaya-Morgenthaler-Staudte (KMS) approach. Difference bars filled in red represent bins where statistically significant differences were found by consensus between the Storer-Kim (SK) and KMS methods, using α = 0.05 with Bonferroni correction, as implemented in the WRS package of Rand Wilcox in statistical package R; otherwise, the bars remain unfilled. The charge values were computed by using a modified BioPerl package.


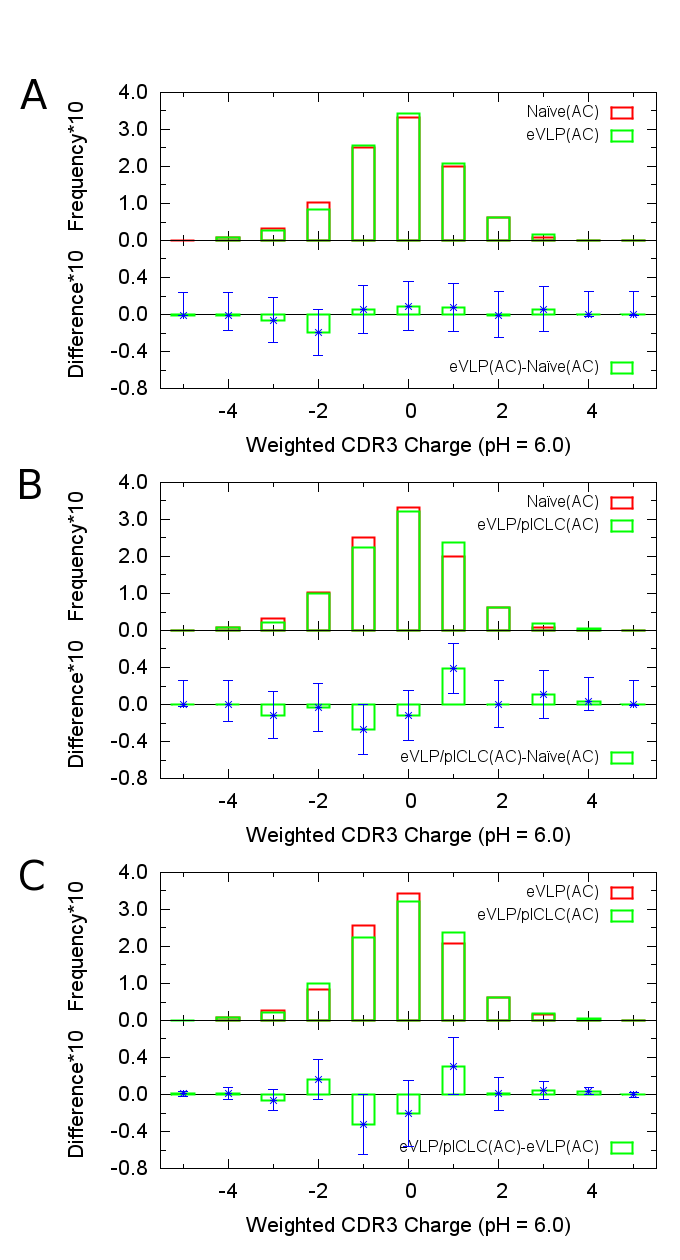


**Figure S2.** Pair-wise comparisons of distributions of clonotype-weighted charge of the CDR3 loop at pH = 6. A) eVLP group relative to Naïve group. B) eVLP/pICLC group relative to Naïve group. C) eVLP/pICLC group relative to eVLP group. All clonotypes (AC) were used in these comparisons. In each subplot, the top pane shows normalized histograms (bin size: 1 charge unit) representing the corresponding distributions; the bottom pane shows the differences between the corresponding histograms, along with confidence intervals, derived by using the Kulinskaya-Morgenthaler-Staudte (KMS) approach. Difference bars filled in red represent bins where statistically significant differences were found by consensus between the Storer-Kim (SK) and KMS methods, using α = 0.05 with Bonferroni correction, as implemented in the WRS package of Rand Wilcox in statistical package R; otherwise, the bars remain unfilled. The charge values were computed by using a modified BioPerl package.


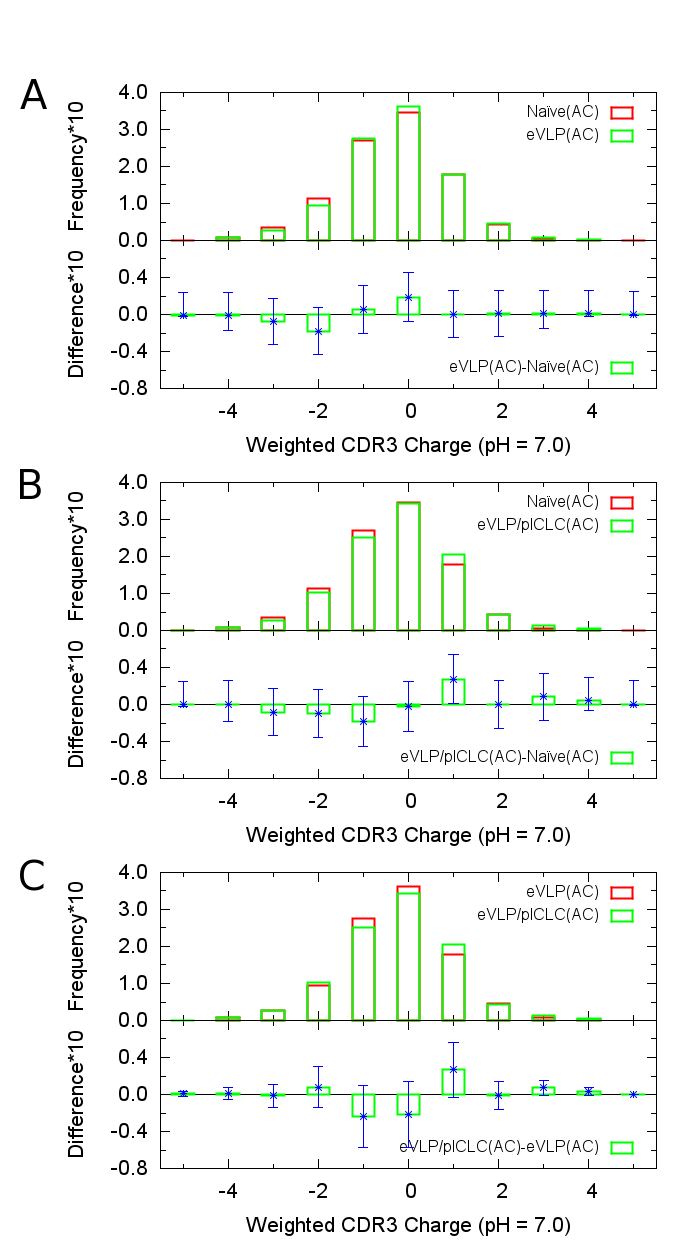


**Figure S3.** Pair-wise comparisons of distributions of clonotype-weighted charge of the CDR3 loop at pH = 7. A) eVLP group relative to Naïve group. B) eVLP/pICLC group relative to Naïve group. C) eVLP/pICLC group relative to eVLP group. All clonotypes (AC) were used in these comparisons. In each subplot, the top pane shows normalized histograms (bin size: 1 charge unit) representing the corresponding distributions; the bottom pane shows the differences between the corresponding histograms, along with confidence intervals, derived by using the Kulinskaya-Morgenthaler-Staudte (KMS) approach. Difference bars filled in red represent bins where statistically significant differences were found by consensus between the Storer-Kim (SK) and KMS methods, using α = 0.05 with Bonferroni correction, as implemented in the WRS package of Rand Wilcox in statistical package R; otherwise, the bars remain unfilled. The charge values were computed by using a modified BioPerl package.


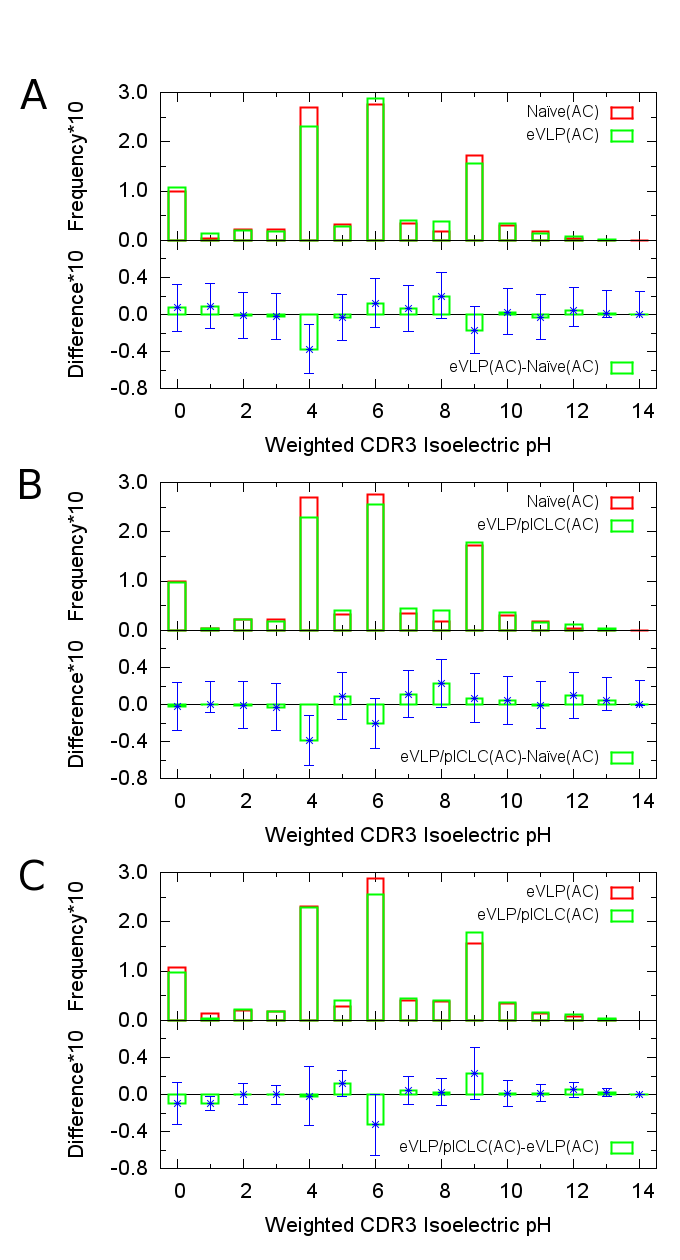


**Figure S4.** Pair-wise comparisons of distributions of clonotype-weighted isoelectric pH of the CDR3 loop. A) eVLP group relative to Naïve group. B) eVLP/pICLC group relative to Naïve group. C) eVLP/pICLC group relative to eVLP group. All clonotypes (AC) were used in these comparisons. In each subplot, the top pane shows normalized histograms (bin size: 1 pH unit) representing the corresponding distributions; the bottom pane shows the differences between the corresponding histograms, along with confidence intervals, derived by using the Kulinskaya-Morgenthaler-Staudte (KMS) approach. Difference bars filled in red represent bins where statistically significant differences were found by consensus between the Storer-Kim (SK) and KMS methods, using α = 0.05 with Bonferroni correction, as implemented in the WRS package of Rand Wilcox in statistical package R; otherwise, the bars remain unfilled. The isoelectric pH values were computed by using a modified BioPerl package.


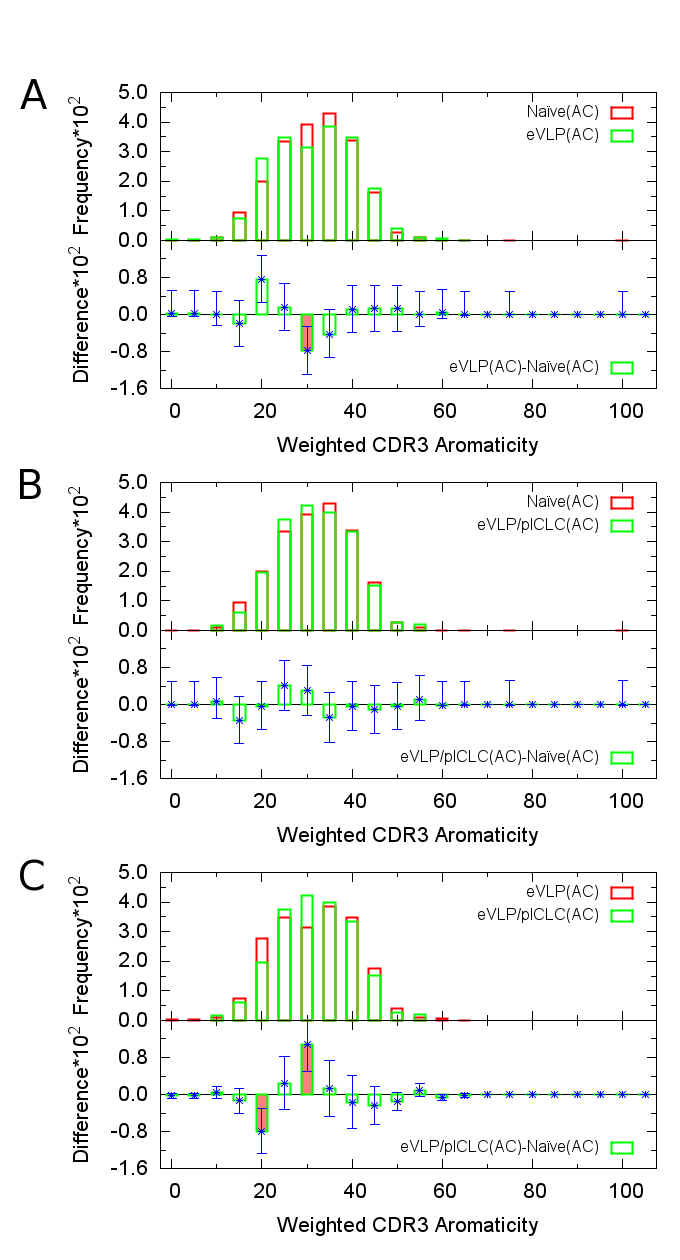


**Figure S5.** Pair-wise comparisons of distributions of clonotype-weighted aromaticity of the CDR3 loop. A) eVLP group relative to Naïve group. B) eVLP/pICLC group relative to Naïve group. C) eVLP/pICLC group relative to eVLP group. All clonotypes (AC) were used in these comparisons. In each subplot, the top pane shows normalized histograms (bin size: 10 units of aromaticity) representing the corresponding distributions; the bottom pane shows the differences between the corresponding histograms, along with confidence intervals, derived by using the Kulinskaya-Morgenthaler-Staudte (KMS) approach. Difference bars filled in red represent bins where statistically significant differences were found by consensus between the Storer-Kim (SK) and KMS methods, using α = 0.05 with Bonferroni correction, as implemented in the WRS package of Rand Wilcox in statistical package R; otherwise, the bars remain unfilled. The aromaticity values were computed by using the BioPerl package.


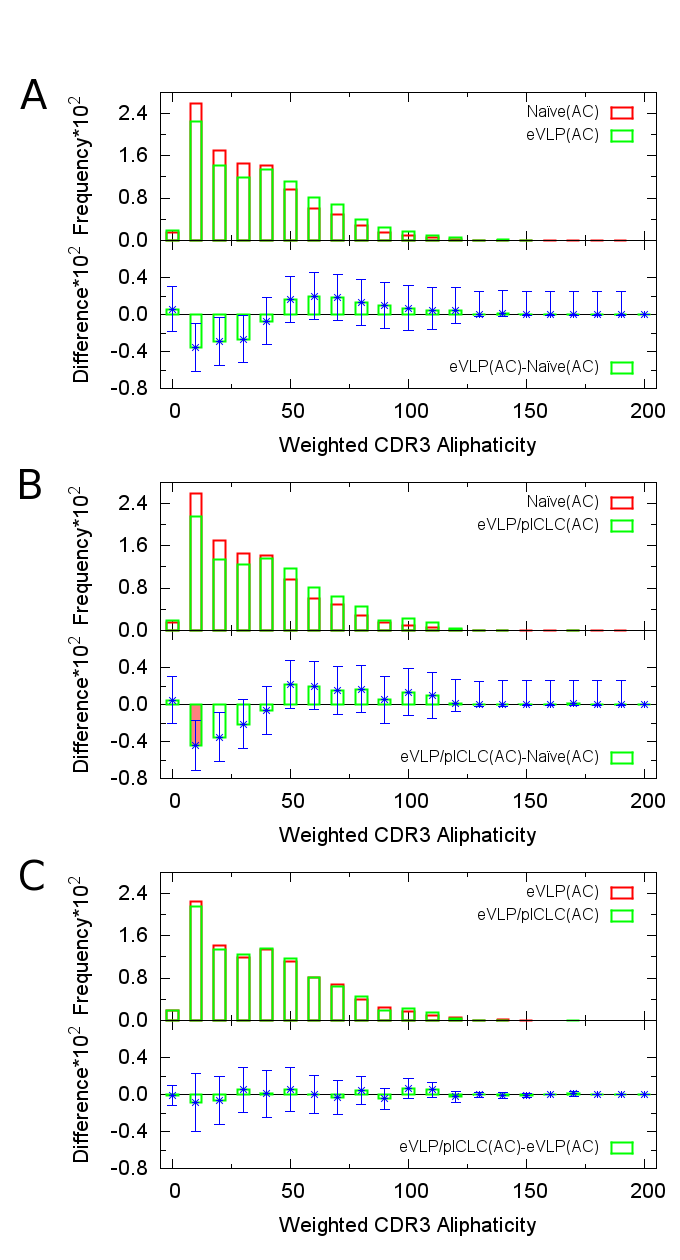


**Figure S6.** Pair-wise comparisons of distributions of clonotype-weighted aliphaticity of the CDR3 loop. A) eVLP group relative to Naïve group. B) eVLP/pICLC group relative to Naïve group. C) eVLP/pICLC group relative to eVLP group. All clonotypes (AC) were used in these comparisons. In each subplot, the top pane shows normalized histograms (bin size: 10 units of aliphaticity) representing the corresponding distributions; the bottom pane shows the differences between the corresponding histograms, along with confidence intervals, derived by using the Kulinskaya-Morgenthaler-Staudte (KMS) approach. Difference bars filled in red represent bins where statistically significant differences were found by consensus between the Storer-Kim (SK) and KMS methods, using α = 0.05 with Bonferroni correction, as implemented in the WRS package of Rand Wilcox in statistical package R; otherwise, the bars remain unfilled. The aliphaticity values were computed by using the BioPerl package.


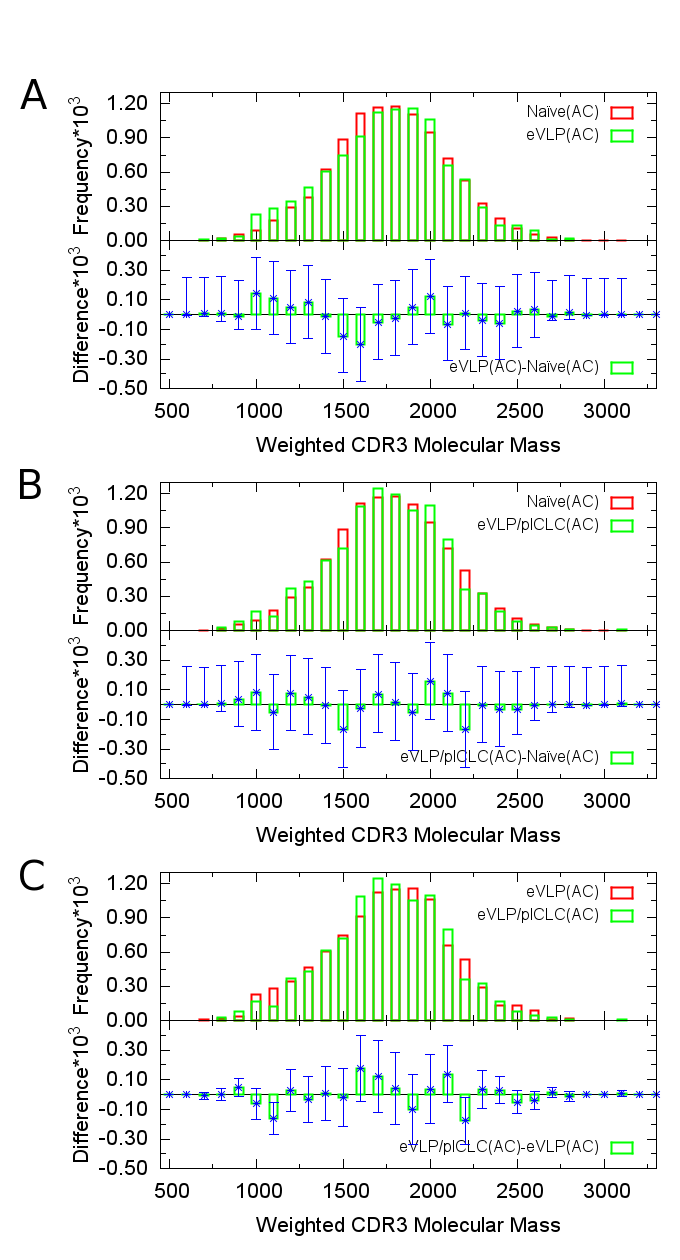


**Figure S7.** Pair-wise comparisons of distributions of clonotype-weighted molecular mass of the CDR3 loop. A) eVLP group relative to Naïve group. B) eVLP/pICLC group relative to Naïve group. C) eVLP/pICLC group relative to eVLP group. All clonotypes (AC) were used in these comparisons. In each subplot, the top pane shows the normalized histograms (bin size: 100 units of molecular mass) representing the corresponding distributions; the bottom pane shows the differences between the corresponding histograms, along with confidence intervals, derived by using the Kulinskaya-Morgenthaler-Staudte (KMS) approach. Difference bars filled in red represent bins where statistically significant differences were found by consensus between the Storer-Kim (SK) and KMS methods, using α = 0.05 with Bonferroni correction, as implemented in the WRS package of Rand Wilcox in statistical package R; otherwise, the bars remain unfilled. The molecular mass values were computed by using the BioPerl package.


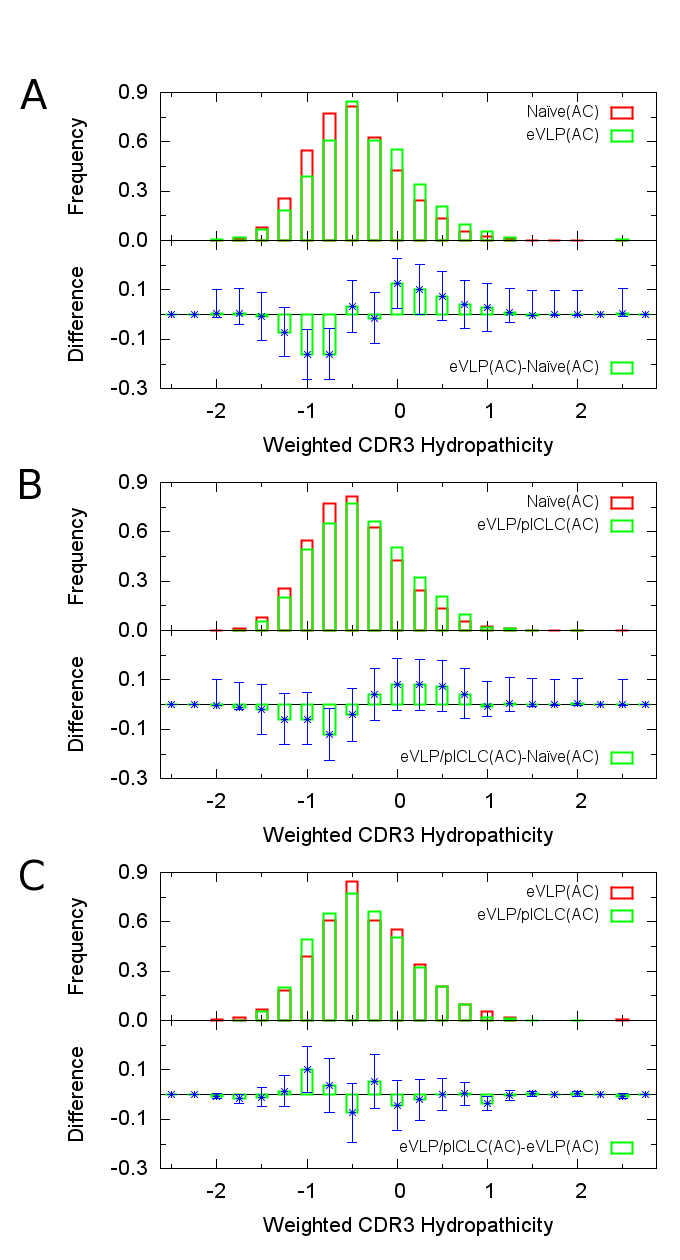


**Figure S8.** Pair-wise comparisons of distributions of clonotype-weighted hydropathicity of the CDR3 loop. A) eVLP group relative to Naïve group. B) eVLP/pICLC group relative to Naïve group. C) eVLP/pICLC group relative to eVLP group. All clonotypes (AC) were used in these comparisons. In each subplot, the top pane shows the normalized histograms (bin size: 0.25 units of hydropathicity) representing the corresponding distributions; the bottom pane shows the differences between the corresponding histograms, along with confidence intervals, derived by using the Kulinskaya-Morgenthaler-Staudte (KMS) approach. Difference bars filled in red represent bins where statistically significant differences were found by consensus between the Storer-Kim (SK) and KMS methods, using α = 0.05 with Bonferroni correction, as implemented in the WRS package of Rand Wilcox in statistical package R; otherwise, the bars remain unfilled. The hydropathicity values were computed by using the BioPerl package.


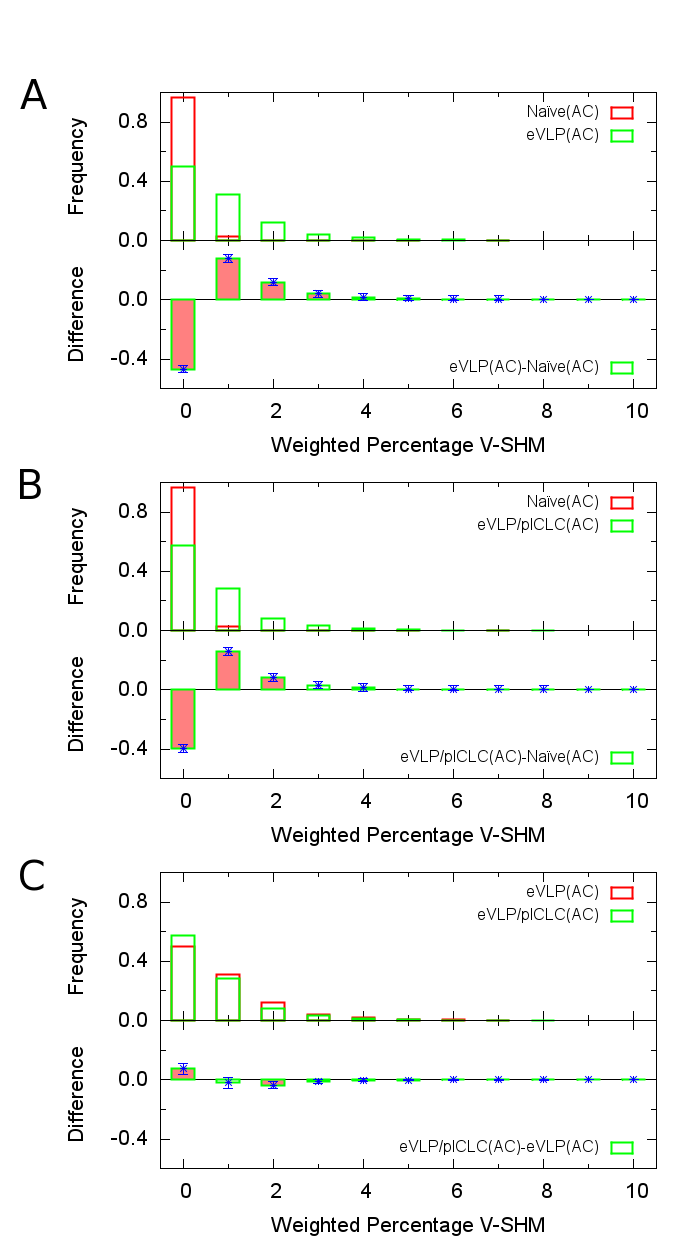


**Figure S9.** Pair-wise comparisons of distributions of clonotype-weighted V-SHM percentage. A) eVLP group relative to Naïve group. B) eVLP/pICLC group relative to Naïve group. C) eVLP/pICLC group relative to eVLP group. All clonotypes (AC) were used in these comparisons. In each subplot, the top pane shows the normalized histograms (bin size: 1% mutation) representing the corresponding distributions; the bottom pane shows the differences between the corresponding histograms, along with confidence intervals, derived by using the Kulinskaya-Morgenthaler-Staudte (KMS) approach. Difference bars filled in red represent bins where statistically significant differences were found by consensus between the Storer-Kim (SK) and KMS methods, using α = 0.05 with Bonferroni correction, as implemented in the WRS package of Rand Wilcox in statistical package R; otherwise, the bars remain unfilled. The V-SHM percentage values were computed by using the BioPerl package.


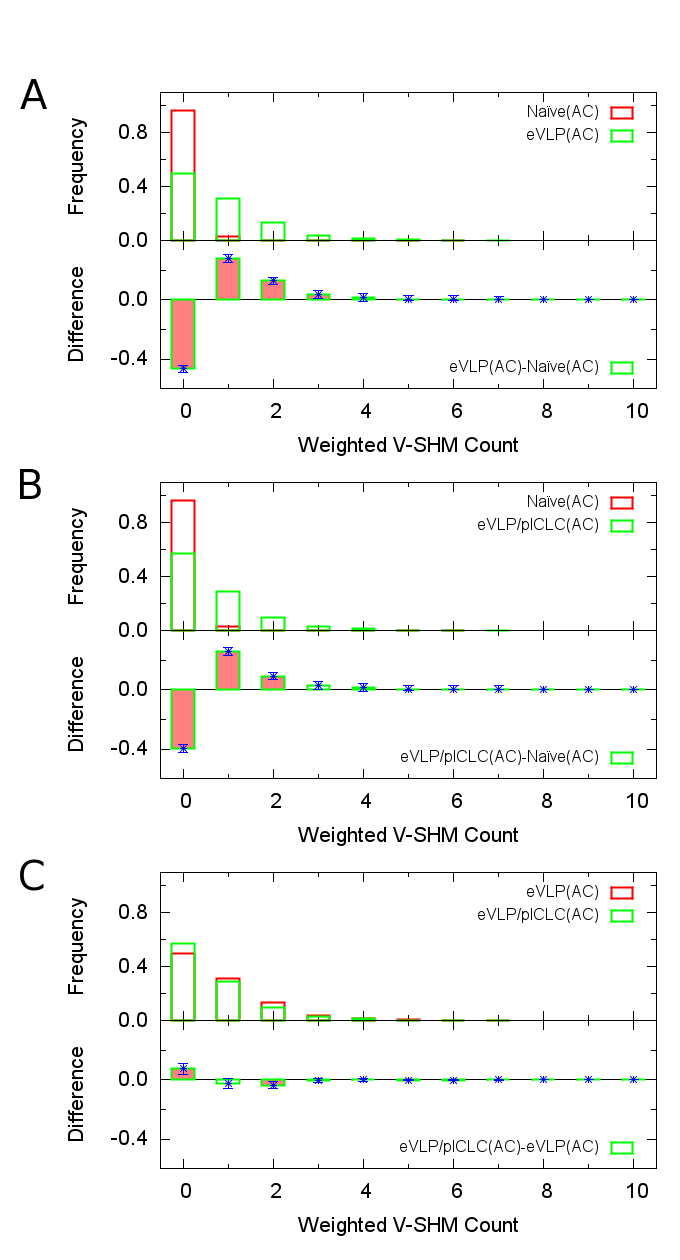


**Figure S10.** Pair-wise comparisons of distributions of clonotype-weighted V-SHM count. A) eVLP group relative to Naïve group. B) eVLP/pICLC group relative to Naïve group. C) eVLP/pICLC group relative to eVLP group. All clonotypes (AC) were used in these comparisons. In each subplot, the top pane shows the normalized histograms (bin size: 1 mutation) representing the corresponding distributions; the bottom pane shows the differences between the corresponding histograms, along with confidence intervals, derived by using the Kulinskaya-Morgenthaler-Staudte (KMS) approach. Difference bars filled in red represent bins where statistically significant differences were found by consensus between the Storer-Kim (SK) and KMS methods, using α = 0.05 with Bonferroni correction, as implemented in the WRS package of Rand Wilcox in statistical package R; otherwise, the bars remain unfilled. The V-SHM counts were computed by using the BioPerl package.


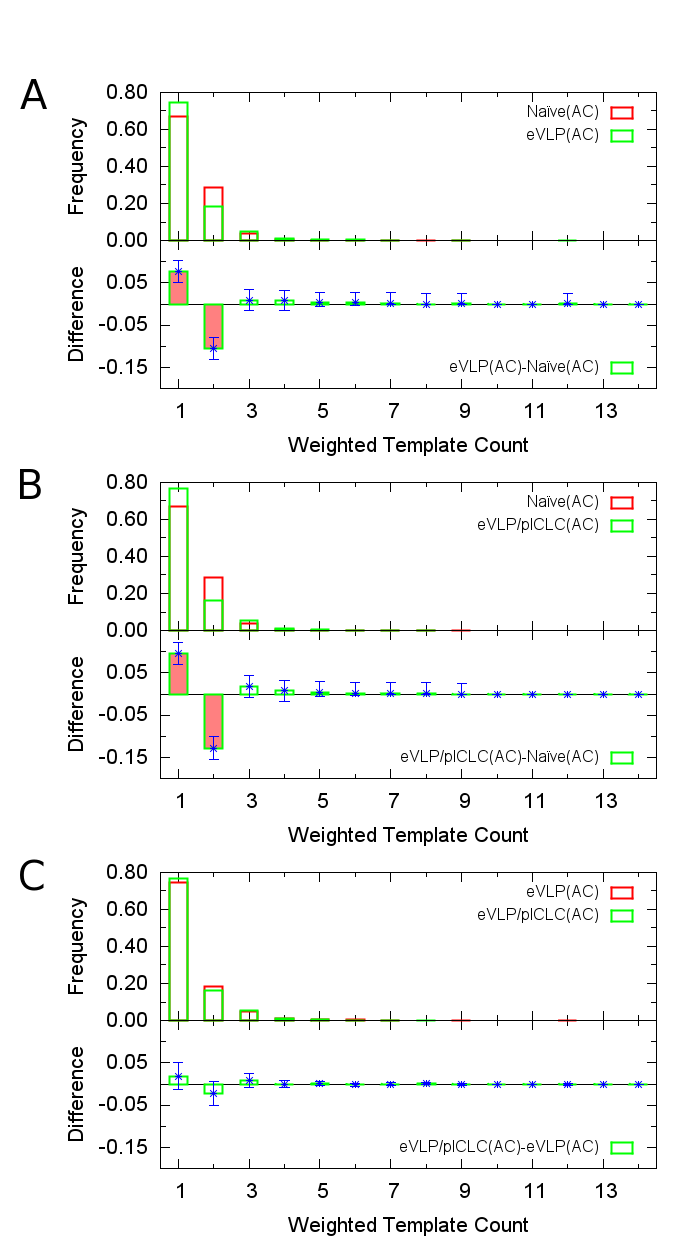


**Figure S11.** Pair-wise comparisons of distributions of clonotype-weighted template count. A) eVLP group relative to Naïve group. B) eVLP/pICLC group relative to Naïve group. C) eVLP/pICLC group relative to eVLP group. All clonotypes (AC) were used in these comparisons. In each subplot, the top pane shows the normalized histograms (bin size: 1 template count) representing the corresponding distributions; the bottom pane shows the differences between the corresponding histograms, along with confidence intervals, derived by using the Kulinskaya-Morgenthaler-Staudte (KMS) approach. Difference bars filled with red represent bins where statistically significant differences were found by consensus between the Storer-Kim (SK) and KMS methods, using α = 0.05 with Bonferroni correction, as implemented in the WRS package of Rand Wilcox in statistical package R; otherwise, the bars remain unfilled. The weighted template counts were computed from template counts of individual sequences, as provided by Adaptive Biotechnology.


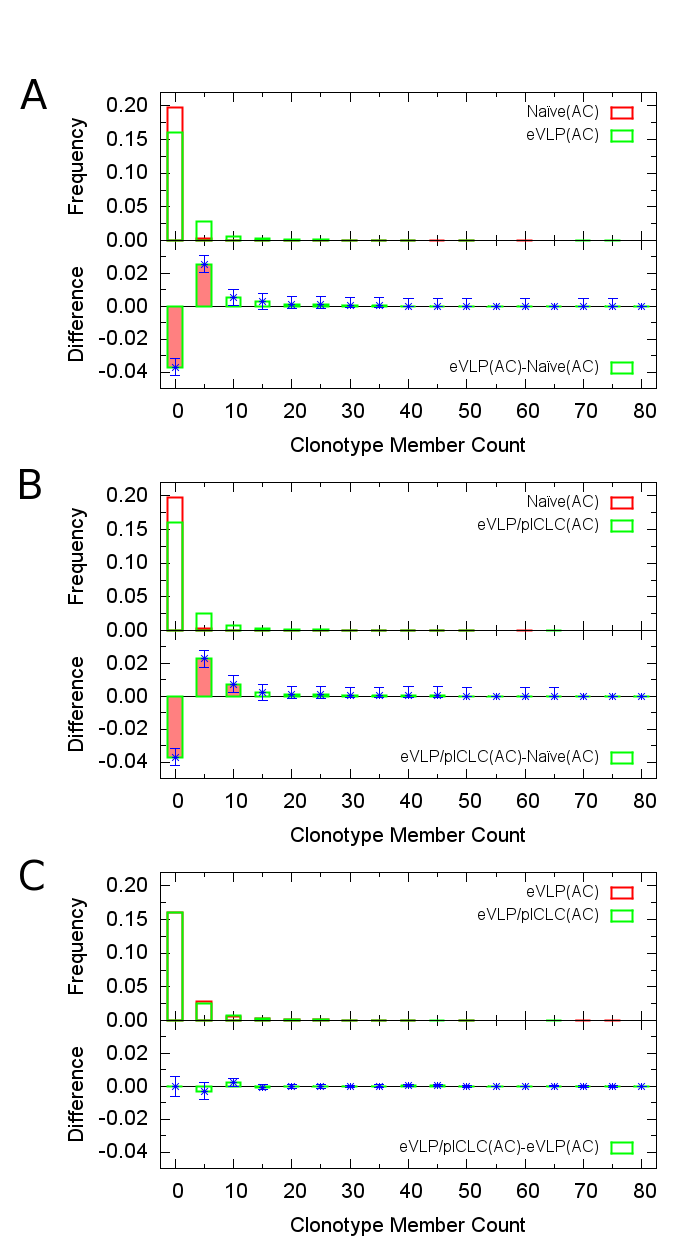


**Figure S12.** Pair-wise comparisons of distributions of clonotype member count. A) eVLP group relative to Naïve group. B) eVLP/pICLC group relative to Naïve group. C) eVLP/pICLC group relative to eVLP group. All clonotypes (AC) were used in these comparisons. In each subplot, the top pane shows the normalized histograms (bin size: 5 members) representing the corresponding distributions; the bottom pane shows the differences between the corresponding histograms, along with confidence intervals, derived by using the Kulinskaya-Morgenthaler-Staudte (KMS) approach. Difference bars filled in red represent bins where statistically significant differences were found by consensus between the Storer-Kim (SK) and KMS methods, using α = 0.05 with Bonferroni correction, as implemented in the WRS package of Rand Wilcox in statistical package R; otherwise, the bars remain unfiled. The member counts were computed from the clonotype clustering method described in the main text.

## Supplementary Tables

Table S1. Summary of immunosequencing data and clonality scores

|  | **All sequences** | | **Productive sequences** | | | **Partitioned AC clonotypes** | | | | **Clonality** | |
| --- | --- | --- | --- | --- | --- | --- | --- | --- | --- | --- | --- |
|  | NA | NA | NA | NA | AC | SC | | BC | | NA | AC |
|  | M | T | M | T | M | M | T | M | T | T | M |
| **Groups** | | | | | | | | | | | |
| Naïve | 50,464 | 68,562 | 35,509 | 48,582 | 31,817 | 29,544 | 40,595 | 2273 | 7,987 | 0.01 | 0.01 |
| eVLP | 5,331 | 8,481 | 3,516 | 5,917 | 1,410 | 949 | 1,181 | 461 | 4,736 | 0.07 | 0.10 |
| eVLP/pICLC | 5,532 | 9,191 | 3,581 | 6,246 | 1,328 | 936 | 1,123 | 392 | 5,123 | 0.07 | 0.11 |
| **Subjects** | | | | | | | | | | | |
| **Naïve** | | | | | | | | | | | |
| S1 | 25,379 | 34,921 | 17,938 | 24,885 | 16,031 | 14,845 | 20,690 | 1,186 | 4,195 | 0.01 | 0.01 |
| S2 | 25,085 | 33,641 | 17,571 | 23,697 | 15,786 | 14,699 | 19,905 | 1,087 | 3,792 | 0.01 | 0.01 |
| **eVLP** | | | | | | | | | | | |
| S3 | 3,667 | 6,078 | 2,451 | 4,274 | 908 | 598 | 745 | 310 | 3,529 | 0.08 | 0.11 |
| S4* | 765 | 1,021 | 519 | 738 | 301 | 222 | 283 | 79 | 455 | 0.04 | 0.06 |
| S5 | 899 | 1,382 | 546 | 905 | 201 | 129 | 153 | 72 | 752 | 0.08 | 0.12 |
| **eVLP/pICLC** | | | | | | | | | | | |
| S6 | 2,638 | 4,639 | 1,758 | 3,329 | 620 | 435 | 522 | 185 | 2,807 | 0.10 | 0.13 |
| S7 | 2,481 | 3,975 | 1,590 | 2,623 | 561 | 376 | 458 | 185 | 2,165 | 0.06 | 0.12 |
| S8 | 413 | 577 | 233 | 294 | 147 | 125 | 143 | 22 | 151 | 0.04 | 0.10 |

NA: all unique sequences before clustering; AC: all unique clonotypes after clonotype clustering; SC: solitary clonotypes, i.e., clonotypes with a single member; BC: branched clonotypes, i.e., clonotypes with more than one member; M: unique sequence based counts; T: template-based counts; S1–S8: subjects ordered by the number of their AC clonotypes within each group; asterisk (*): subject that would have been ordered differently if all unique productive sequences were used to order subjects instead of clonotypes. Clonality scores, as defined by Adaptive Biotechnology (i.e., clonality = 1 – normalized entropy), were computed by using productive sequences only.

Table S2. Additional indices of diversity for productive immunoglobulin repertoires

|  | **Diversity (Gini index)** | | | **Normalized Entropy** | | | **Entropy** | | |
| --- | --- | --- | --- | --- | --- | --- | --- | --- | --- |
|  | NA | AC | AC | NA | AC | AC | NA | AC | AC |
|  | T | M | T | T | M | T | T | M | T |
| **Groups** | | | | | | | | | |
| Naïve | 0.10 | 0.05 | 0.13 | 0.99 | 0.99 | 0.98 | 15.00 | 14.82 | 14.71 |
| eVLP | 0.18 | 0.26 | 0.33 | 0.93 | 0.91 | 0.84 | 11.00 | 9.47 | 8.83 |
| eVLP/pICLC | 0.20 | 0.28 | 0.35 | 0.93 | 0.89 | 0.83 | 10.95 | 9.24 | 8.60 |
| **Subjects** | | | | | | | | | |
| **Naïve** | | | | | | | | | |
| S1 | 0.10 | 0.05 | 0.13 | 0.99 | 0.99 | 0.98 | 14.01 | 13.83 | 13.72 |
| S2 | 0.10 | 0.05 | 0.12 | 0.99 | 0.99 | 0.98 | 13.99 | 13.81 | 13.71 |
| **eVLP** | | | | | | | | | |
| S3 | 0.19 | 0.27 | 0.34 | 0.92 | 0.89 | 0.82 | 10.38 | 8.74 | 8.08 |
| S4* | 0.13 | 0.18 | 0.24 | 0.96 | 0.94 | 0.91 | 8.69 | 7.74 | 7.46 |
| S5 | 0.18 | 0.27 | 0.33 | 0.93 | 0.88 | 0.79 | 8.42 | 6.74 | 6.08 |
| **eVLP/pICLC** | | | | | | | | | |
| S6 | 0.22 | 0.28 | 0.36 | 0.90 | 0.87 | 0.79 | 9.74 | 8.10 | 7.32 |
| S7 | 0.18 | 0.28 | 0.34 | 0.94 | 0.88 | 0.83 | 9.96 | 8.03 | 7.60 |
| S8 | 0.10 | 0.17 | 0.23 | 0.96 | 0.90 | 0.86 | 7.57 | 6.47 | 6.16 |

NA: all unique sequences before clustering; AC: all unique clonotypes after clonotype clustering; M: unique sequence based counts; T: template-based counts; S1–S8: subjects ordered by the number of their AC clonotypes within each group; asterisk (*): subject that would have been ordered differently if all unique productive sequences were used to order subjects instead of clonotypes. All diversity measures were computed by using productive sequences only.

Table S3. Sensitivity analysis of the IGHV-family usage

| **Down-sampling condition** | **Vaccine Condition** | **Vaccinated Group AC Repertoire Size (%)** | **Control Group AC Repertoire Size (%)** | **Percentage outcomes based on 10 comparisons** | | | | | | |
| --- | --- | --- | --- | --- | --- | --- | --- | --- | --- | --- |
|  |  |  |  | **V8 (TP)** | **V1 (FP)** | **V2 (FP)** | **V4 (FP)** | **V6 (FP)** | **V9 (FP)** | **V10 (FP)** |
| Decreasing sample size of control group | eVLP | 1410 (100%) | 7954 (25%) | 100 |  |  |  |  |  |  |
|  | eVLP/pICLC | 1328 (100%) | 7954 (25%) | 100 |  |  |  |  |  |  |
|  | eVLP | 1410 (100%) | 3182 (10%) | 100 |  | 10 |  |  |  |  |
|  | eVLP/pICLC | 1328 (100%) | 3182 (10%) | 100 |  |  |  |  |  |  |
|  | eVLP | 1410 (100%) | 1590 (5%) | 100 |  | 70 | 10 | 10 |  | 70 |
|  | eVLP/pICLC | 1328 (100%) | 1590 (5%) | 100 |  | 50 |  |  | 20 | 50 |
|  | eVLP | 1410 (100%) | 318 (1%) | 70 |  |  |  |  |  |  |
|  | eVLP/pICLC | 1328 (100%) | 318 (1%) | 60 |  |  |  |  |  |  |
| Decreasing sample size of vaccinated group | eVLP | 705 (50%) | 31817 (100%) | 100 |  |  |  |  |  |  |
|  | eVLP/pICLC | 664 (50%) | 31817 (100%) | 90 |  |  |  |  |  |  |
|  | eVLP | 352 (25%) | 31817 (100%) | 50 |  |  |  |  |  |  |
|  | eVLP/pICLC | 332 (25%) | 31817 (100%) | 40 | 10 |  |  |  |  |  |
| Balanced sample size of control and vaccinated groups | eVLP | 1200 (85.10%) | 1200 (3.77%) | 100 |  | 40 | 20 | 20 |  | 40 |
|  | eVLP/pICLC | 1200 (90.36%) | 1200 (3.77%) | 100 |  | 30 |  |  | 20 | 10 |
|  | eVLP | 800 (56.74%) | 800 (2.51%) | 100 |  |  | 10 |  |  | 30 |
|  | eVLP/pICLC | 800 (60.24%) | 800 (2.51%) | 100 |  | 20 |  |  | 20 |  |
|  | eVLP | 400 (28.37%) | 400 (1.26%) | 90 |  | 10 |  |  |  |  |
|  | eVLP/pICLC | 400 (30.12%) | 400 (1.26%) | 80 |  |  |  |  | 10 |  |

AC – all clonotypes. TP and FP refer to true positive and false positive, respectively, defined relative to the SK/KMS analysis results for full AC. Down-sampling was performed using in-house Perl script interfaced with R (sample.int function for random samples without replacement). We drew 10 samples, using random seed 12345, for both vaccinated and unvaccinated groups when down-sampling only a single group. When down-sampling both control and vaccinated groups to match sample size, we used seeds 12345 and 54321 for the vaccinated and unvaccinated groups, respectively. The numbers in the TP and FP columns show the SK/KMS testing outcome percentage based on 10 independent comparisons.

**
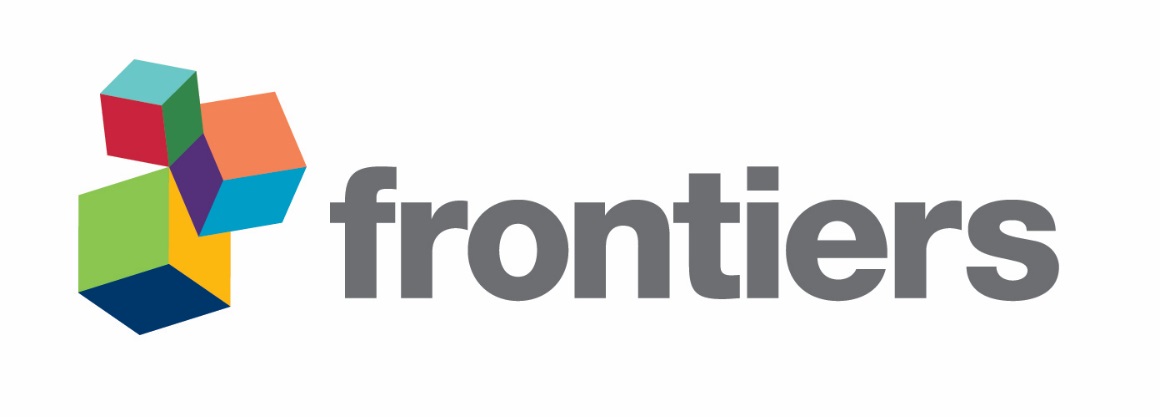
**
